# Untargeted metabolomic analyses support the main phylogenetic groups of the common plant-associated *Alternaria* fungi isolated from grapevine (*Vitis vinifera*)

**DOI:** 10.1038/s41598-023-46020-3

**Published:** 2023-11-07

**Authors:** Anna Molnár, Dániel G. Knapp, Miklós Lovas, Gergő Tóth, Imre Boldizsár, Kálmán Zoltán Váczy, Gábor M. Kovács

**Affiliations:** 1 Centre for Research and Development, Eszterházy Károly Catholic University, Leányka utca 6, Eger, 3300 Hungary; 2 Department of Plant Anatomy, Institute of Biology, Eötvös Loránd University, Pázmány Péter sétány 1/C, Budapest, 1117 Hungary; 3 Hevesy György PhD School of Chemistry, Institute of Chemistry, ELTE Eötvös Loránd University, Budapest, H-1117 Hungary; 4 Department of Pharmaceutical Chemistry, Semmelweis University, Hőgyes Endre U. 9, Budapest, 1092 Hungary; 5 Department of Pharmacognosy, Semmelweis University, Üllői út 26, Budapest, 1085 Hungary; 6 Plant Protection Institute, Centre for Agricultural Research, Budapest, 1525 Hungary; 7 Present Address: Department of Forestry and Wood Technology, Linnaeus University, Växjö, Sweden

**Keywords:** Fungi, Microbiome

## Abstract

*Alternaria*, a cosmopolitan fungal genus is a dominant member of the grapevine (*Vitis vinifera*) microbiome. Several *Alternaria* species are known to produce a variety of secondary metabolites, which are particularly relevant to plant protection and food safety in field crops. According to previous findings, the majority of *Alternaria* species inhabiting grapevine belong to *Alternaria* sect. *Alternaria*. However, the phylogenetic diversity and secondary metabolite production of the distinct *Alternaria* species has remained unclear. In this study, our aim was to examine the genetic and metabolic diversity of endophytic *Alternaria* isolates associated with the above-ground tissues of the grapevine. Altogether, 270 *Alternaria* isolates were collected from asymptomatic leaves and grape clusters of different grapevine varieties in the Eger wine region of Hungary. After analyses of the nuclear ribosomal DNA internal transcribed spacer (ITS) and RNA polymerase second largest subunit (*rpb2*) sequences, 170 isolates were chosen for further analyses. Sequences of the *Alternaria* major allergen gene (*Alt a 1*), endopolygalacturonase (*endoPG*), OPA10-2, and KOG1058 were also included in the phylogenetic analyses. Identification of secondary metabolites and metabolite profiling of the isolates were performed using high-performance liquid chromatography (HPLC)–high-resolution tandem mass spectrometry (HR-MS/MS). The multilocus phylogeny results revealed two distinct groups in grapevine, namely *A*. *alternata* and the *A*. *arborescens* species complex (AASC). Eight main metabolites were identified in all collected *Alternaria* isolates, regardless of their affiliation to the species and lineages. Multivariate analyses of untargeted metabolites found no clear separations; however, a partial least squares-discriminant analysis model was able to successfully discriminate between the metabolic datasets from isolates belonging to the AASC and *A. alternata*. By conducting univariate analysis based on the discriminant ability of the metabolites, we also identified several features exhibiting large and significant variation between *A. alternata* and the AASC. The separation of these groups may suggest functional differences, which may also play a role in the functioning of the plant microbiome.

## Introduction

Grapevine (*V. vinifera*) holds significant economic importance globally, and it is associated with a diverse microbiome. A fungal core microbiome of grapevine, independent of the season and region, has been reported from recent studies using both culture-dependent and culture-independent methods, confirming the predominance of the genera *Alternaria, Aureobasidium*, *Botrytis, Cladosporium*, *Epicoccum*, *Fusarium,* and *Penicillium*^1–11^. According to these studies, members of *Alternaria* sect. *Alternaria* are among the most common endophytes and constitute a dominant fungal consortium in different grapevine varieties. *Alternaria* is a biologically, morphologically, and ecologically diverse fungal genus, including cosmopolitan saprobes, endophytes, pathogens, and causal agents of post-harvest rots, producing substantial economic losses for different agronomic plants^12^. *Alternaria* spp. have been commonly identified based on conidial and colony characteristics, although morphological traits, especially of small-spored species, often overlap^13^. Due to this heterogeneity, the delineation of species is challenging. Recent advances in multilocus phylogeny have accelerated the identification of *Alternaria* species, and the genus has been reorganized into 27 sections based on sequences commonly used in the molecular phylogeny of other fungal genera, including nrDNA SSU, LSU, ITS, β-tubulin, *tef1*, calmodulin, actin, and expanded analyses of sequences of *rpb2*, *endoPG*, *Alt a 1*, *gapdh*, OPA1-3, OPA10-2, KOG1058, and KOG1077^14–25^. Among the 27 phylogenetic sections, section *Alternaria*—closely related to sections *Alternantherae* and *Porri*—consists of the species complex *A. arborescens* (AASC) and 11 phylogenetic species (*A. burnsii*, *A. tomato*, *A. jacinthicola*, *A. iridiaustralis*, *A. eichhorniae*, *A. betae-kenyensis*, *A. gaisen*, *A. alstroemeriae*, *A. longipes*, *A. gossypina,* and *A. alternata*), from which *A. alternata* comprises 35 morphospecies^23^. This genus has gained increasing attention due to its ability to produce a broad spectrum of biologically active secondary metabolites with phytotoxic, cytotoxic, and antimicrobial properties^26^. *Alternaria* species produce more than 250 metabolites, mainly nitrogen-containing compounds, steroids, terpenoids, pyranones, quinones, and phenolics^26^. The major *Alternaria* toxins belong to the chemical groups of dibenzo-α-pyrones and cyclic tetrapeptides^27^. Based on chemical structure, *Alternaria* mycotoxins can be divided into three further classes: perylene (derivatives); tetramic acid derivatives; and the TA1, TA2, TB1, and TB2 toxins from *A. alternata* subspecies *lycopersici*^28^. Qualitative analysis of secondary metabolite production using high-resolution chromatographic techniques has been successfully used as a tool for the segregation and differentiation of *Alternaria*^29–36^. These studies have demonstrated that metabolite profiling can be informative in the grouping of large-spored *Alternaria* isolates but have revealed further ambiguity among small-spored *Alternaria* species.

Culture-independent methods provide a powerful tool for detailed grapevine microbiome studies; although, these methods tend to perform poorly in species-level identification in many fungal groups. The ITS region of the nuclear ribosomal DNA has been commonly used to characterize the diversity and composition of fungal communities in grapevine; however, the taxonomic resolution of this single locus is low in the case of *Alternaria*^37^. The ITS region alone is not adequate for species-level discrimination, and species belonging to *Alternaria* sect. *Alternaria* have been frequently identified in grapevine as *A*. *alternata* (e.g.^10^). Due to their potential taxonomic diversity with distinct functions and metabolite production, gaining information regarding the grapevine-colonizing species of *Alternaria* sect. *Alternaria* would provide important insights.

As our previous results have demonstrated the common presence of diverse *Alternaria* members in the wine grape microbiome^10^, we aimed in this study to reveal the diversity of *Alternaria* colonizing asymptomatic tissues of different cultivars of *V. vinifera* in a historical wine region of Hungary. Our main goal was to collect *Alternaria* isolates, identify the taxonomic identity of the isolates using multilocus molecular phylogenetic analyses, and determine whether there is a correspondence between these groups based on an untargeted chemical analysis of the secondary metabolites produced by those isolates.

## Materials and methods

### Isolation of fungal endophytes

Different parts (leaves, grape clusters, and berries) of several grapevine varieties (*Vitis vinifera* cv. Furmint, cv. Pinot Noir, cv. Merlot, cv. Leányka, cv. Chardonnay, and cv. Kadarka, as well as unidentified cultivars) were sampled between August and September of 2019 from seven vineyards (Nagy-Eged hegy, Hangács, Kőlyuk-tető, Rác-hegy, Cinege, Déllés, and Hajdú-hegy) in different localities of the Eger wine region of Hungary (Supplementary Table 2). The St. Andrea Winery and the Centre for Research and Development at Eszterházy Károly Catholic University granted authorization to collect plant material for our research on *V. vinifera* cv. Furmint, cv. Pinot Noir, cv. Merlot, cv. Leányka, cv. Chardonnay, cv. Kadarka and unidentified cultivars. Sample collection has complied with relevant institutional, national, and international guidelines and legislation. The region is a cool-climate district located in northeastern Hungary, in the southern foreland of the Bükk Mountains^38^. The 22,160-ha area has high oenological value, containing 6000 ha of vineyards. Rhyolite tuff and volcanic rocks are the characteristic bedrock. Variations of brown forest soils containing clay minerals in high quantity are dominant in the area^39^.

In each location, young leaves (fourth from the shoot tip), mature leaves, and grape clusters (separated into berries and rachis/pedicels) were collected and then stored in plastic bags at 4 °C for further processing within 24 h. Endophyte isolation was conducted as described by Knapp et al.^10^, with modifications. Briefly, samples were sliced into 0.5–1-cm segments, soaked in 30% H_2_O_2_ for 1 min, then soaked in 70% ethanol for 1 min, and washed in sterile tap water twice for 2–3 min. Surface-sterilized samples were transferred onto potato dextrose agar (PDA) media (VWR, Germany) and incubated at 25 °C. Within the following 1–7 days, mycelia growing from the plant tissues were transferred to new PDA plates.

### Genomic DNA extraction and molecular characterization

Mycelia were harvested from pure cultures and disrupted and homogenized in a TissueLyser LT (QIAGEN, Germany). Genomic DNA extraction was performed using the NucleoSpin Plant II DNA Isolation Kit (MACHEREY-NAGEL, Germany), following the manufacturer’s instructions. The internal transcribed spacer region (ITS) of the nuclear ribosomal DNA and partial region of the RNA polymerase II second largest subunit (*rpb2*) were amplified with the primer pairs ITS1F, ITS4^40,41^, and RPB2-6F and fRPB2-7cR^42^, respectively. Representative isolates identified by ITS and *rpb2* as members of *Alternaria* sect*. Alternaria* were chosen for further, multilocus sequence analysis. The loci of the *Alternaria* major allergen gene (*Alt a 1*), endopolygalacturonase (*endoPG*), an approximately 800-bp partial sequence of an anonymous noncoding region (OPA10-2), and the eukaryotic orthologous group (KOG) protein locus (KOG1058) were amplified by polymerase chain reaction with the primer pairs Alt-for and Alt-rev^16^, PG3 and PG2b^17^, OPA10-2R and OPA10-2L^17^, and KOG1058F2 and KOG1058R2^23^, respectively. Because amplification of *Alt a 1* failed using the primer pairs Alt-for and Alt-rev, PCR was carried out with the modified primers Alt4-for and Alt4-rev^43^. Reactions were performed in a T100 Thermal Cycler (Bio-Rad, California, USA) in a 50-µL reaction mixture. Phire Hot Start II PCR Master Mix (Thermo Fisher Scientific, Massachusetts, USA) was used for each reaction. PCR conditions consisted of an initial denaturation step at 98 °C for 30 s, followed by 32 cycles of 98 °C for 5 s denaturation at 98 °C for 5 s; the optimal annealing temperature for each primer pair (see Supplementary Table 3) for 5 s; and extension at 72 °C for 30 s, followed by a final extension at 72 °C for 3 min. PCR products were submitted to Eurofins Genomics GmbH for amplicon sequencing (Ebersberg, Germany). Sequence analysis and editing were performed with the PREGAP4 and GAP4 tools of the Staden software package^44^ and deposited in GenBank (OQ931049–OQ931220, OQ973480–OQ974171; Supplementary Table 1). To obtain preliminary identification of the isolates, ITS sequences were blasted against available sequences from public databases using BLASTN searches^45^.

### Phylogenetic analyses

**Figure 1:**
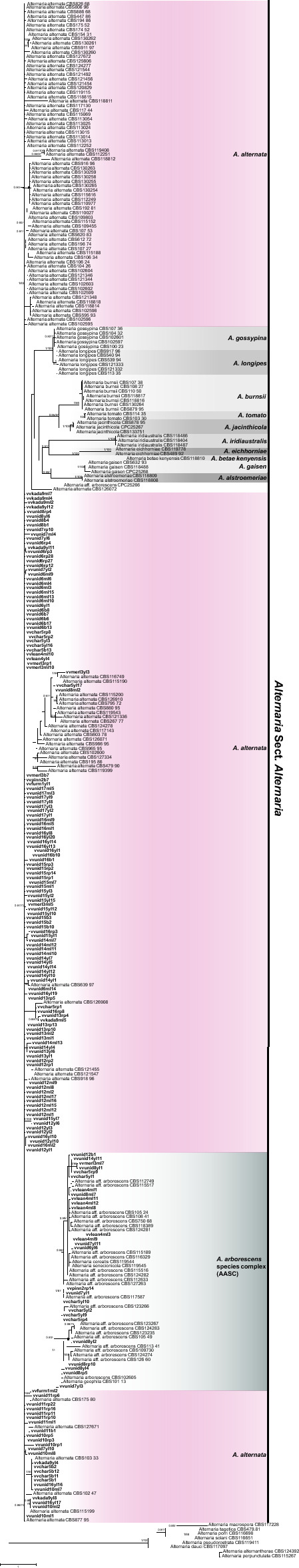
Phylogenetic tree of all *Alternaria* isolates collected from grapevine leaves and clusters and reference *Alternaria* strains from Woudenberg et al.^23^. The 50% majority rule consensus phylogram inferred from Bayesian analysis of the combined dataset of five loci (*rpb2*, ITS, *Alt a 1*, *endoPG*, OPA10-2). Bayesian posterior probabilities (≥ 0.90) are shown before slashes, ML bootstrap support (≥ 70) is shown after slashes. Isolates representing the lineages of *A. alternata* (violet) and *A. arborescens* species complex (AASC, dark green) in this study are shown in bold. Sequences were rooted to *A. alternantherae, A. perpunctulata, A. solani, A. porri, A. tagetica, A. macrospora, A. pseudorostrata* and *A. dauci.* Scale bar indicates 1 expected change per branch.

We combined and aligned the sequences of the different loci with those from representative taxa in GenBank using the online version of MAFFT 7^46^ and following the E-INS-i method. The alignments were examined and edited in MEGA 7^47^. Three multilocus datasets were used for molecular phylogenetic analyses of the isolates and reference strains of *Alternaria* sect. *Alternaria* ^23^ (Supplementary Table 1). For the first dataset, we used sequences of six sequenced loci (ITS, *rpb2*, *Alt a 1*, *endoPG*, OPA10-2, and KOG1058) from our isolates (Supplementary Fig. 1). In the second dataset, five loci (ITS, *rpb2*, *Alt a 1*, *endoPG*, and OPA10-2) from our strains and reference strains of *Alternaria* sect. *Alternaria* sequenced in this study and by Woudenberg et al.^23^ were analyzed (Fig. 1). The third analysis used all seven loci (ITS, *rpb2*, *Alt a 1*, *endoPG*, OPA10-2, *tef1*, and *gapdh*) from the taxa analyzed by Woudenberg et al.^23^, and our isolates were represented by the shared five loci (Supplementary Fig. 2). In the two later phylogenies, *A. alternantherae,* CBS 124,392, and *A. perpunctulata* CBS 115,267 served as an outgroup. In the case of the ITS, *rpb2*, *Alt a 1*, endoPG, OPA10-2, *tef1,* and *gapdh* datasets, the partitions were analyzed separately to examine differences in single-locus phylogenies (Supplementary Fig. 3). Bayesian inference analyses were performed with MRBAYES 3.1.2^48^ using a GTR + G substitution model for the nucleotide partitions. Four Markov chains were run for 10,000,000 generations, sampling every 1,000 generations with a burn-in value set at 4,000 sampled trees. Maximum likelihood (ML) phylogenetic analysis was performed with the RAXMLGUI 1.3 implementation^49,50^. A GTR + G nucleotide substitution model was used for nucleotide partitions with ML estimation of base frequencies. ML bootstrapping (BS) analysis with 1,000 replicates was used to test the support of the branches. Phylogenetic trees were visualized and edited in MEGA 7^47^.

### Metabolite extraction and identification

For metabolite profiling, 170 out of the 173 isolates (three *A. alternata* isolates were lost before these analyses) were subcultured simultaneously and grown in three replicates in Petri dishes (60 mm × 15 mm) on PDA medium (VWR, Hungary) at room temperature in the dark for 21 days. The entire culture-containing PDA medium and the fungal mycelium grown on it were lyophilized and pulverized. Solvents applied in the extraction analysis of metabolites, such as acetonitrile, distilled water, formic acid, and methanol (Reanal, Hungary), were all of the analytical reagent grade of the highest purity available. Aliquots of the powdered cultures (20.0 mg) were extracted with 5.0 mL of methanol in 25 mL screw-capped vials at 60 °C for 30 min. The insoluble, centrifuged material was subsequently re-extracted in the same way. The supernatants were combined to prepare 10.0 mL extracts.

To identify compounds present in extracts of *Alternaria* isolates, a Dionex Ultimate 3000 UHPLC system (3000RS diode array detector [DAD], TCC-3000RS column thermostat, HPG-3400RS pump, SRD-3400 solvent rack degasser, WPS-3000TRS autosampler), connected to an Orbitrap Q Exactive Focus Mass Spectrometer equipped with electrospray ionization (ESI) (Thermo Fischer Scientific, Waltham, MA, USA) was used. High-performance liquid chromatography (HPLC) separations were performed on a Kinetex C18 column (75 × 3 mm; 2.6 μm) (Phenomenex, USA). The eluent A consisted of 0.1% v/v formic acid in water, and eluent B, a mixture of acetonitrile and water in an 80:20, v/v ratio, containing 0.1% v/v formic acid. A linear gradient was applied with initial conditions of 20% B at 0.0 min, reaching 90% B at 12.0 min. The flow rate was maintained at 0.3 mL/min, the temperature was set to 25 °C, and a 5.0 μL volume was injected. The ESI source was operated in positive and negative ionization modes (switching mode). Fragmentations were performed by the data-independent acquisition method using isolation widths of 100–300 m*/z*, 295–500 m*/z*, 495–700 m*/z*, and 695–800 m*/z* and collision energies of 15, 30, and 45 eV. Operation parameters were optimized automatically by the built-in software as follows: spray voltage, 3500 V ( +); capillary temperature, 256 °C; sheath-, auxiliary-, and spare-gases (N_2_): 47.50, 11.25, and 2.25 arbitrary units, respectively. The resolutions of the full scans and MS/MS scans were 70,000 and 35,000, respectively. The full MS scanning range was 100–1000 m*/z* units. UV spectra were recorded between 250 and 600 nm, and UV chromatograms were plotted as summed signal intensities measured in this wavelength range.

### Untargeted mass spectrometry (MS) data processing

Untargeted MS data processing was performed using MZmine 3 metabolomics software^51^. From each raw data file (.RAW) acquired in LC–MS analysis, positive and negative MS1 scan data were extracted into separate NetCDF files. As the MS data were recorded as centroid, a noise-filtering and mass-picking algorithm was used with an intensity threshold of 100,000, followed by a chromatogram building and deconvoluting step performed by the ADAP module^52^. The resulting feature lists (distinct RT-*m/z* pairs) were deisotoped and aligned between samples using the RANSAC algorithm^53^. Features not detected in all of the replicates of a sample were eliminated from the aligned feature list, which was then exported in .CSV format for further statistical analysis.

### Statistical analyses of untargeted MS data

The positive and negative MS data were separately subjected to statistical analysis. In the metabolomic dataset, each feature’s abundance was expressed as peak area, and the data were centered and scaled to achieve zero means and unit variances. Principal component analysis (PCA) was performed as a preliminary analysis using the R package *mixOmics*^54^, followed by a partial least squares discriminant analysis (PLS-DA) using the same package. To evaluate the model’s performance, a three-fold cross-validation was performed, repeated ten times. Welch’s *t*-tests, Mann–Whitney–Wilcoxon tests, and receiver operating characteristic (ROC) analyses were conducted to assess the discriminant ability of each feature. Additionally, fold change and Z-factor were calculated using the R package *imageHTS*. Area under the ROC curve versus Welch’s *p*-value combined with a Z-factor plot was constructed based on the technique of Broadhurst et al.^55^. Heatmaps were generated using the *ComplexHeatmap* R package.

## Results

### Endophyte isolation

In this study, more than 450 plant samples were collected from different grapevine cultivars characteristic of the area of the Eger wine region of Hungary. We collected a total of 570 fungal endophytes after isolation from surface-sterilized young and mature healthy leaves and grape clusters of *V. vinifera* (Supplementary Table 2). Based on the analysis of the ITS sequences, the isolates belonged to several taxa, from which we identified 270 isolates representing species within *Alternaria* sect. *Alternaria*. Common members of the grapevine mycobiome were also detected, including *Aureobasidium pullulans* (28 isolates), *Stemphylium vesicarium* (12) and *Botrytis cinerea* (12), which were present in the highest number among the grapevine-associated isolates (data not shown). According to the groups resulting from phylogenetic analysis of the *rpb2* sequences of the 270 *Alternaria* isolates, 173 representatives were chosen for further multilocus molecular phylogenetic analyses. From these isolates, four genomic loci (*Alt a 1*, *endoPG*, OPA10-2, and KOG1058) in addition to the ITS and *rpb2* sequences were amplified and sequenced. Amplification of *Alt a 1* and OPA10-2 was successful for all 173 isolates; 25 isolates failed for *endoPG,* and 52 failed for KOG1058 (Supplementary Table 1).

### Molecular phylogeny

In the six-locus phylogeny of the 173 isolates, nearly half (74) of the isolates exhibited almost identical sequences and represented one major clade. One distinct, well-supported (B-PP = 1, ML-BS = 96) clade comprised 26 isolates (Supplementary Fig. S1). In the five-locus phylogeny, our isolates from grapevine were analyzed together with reference sequences^23^, which served as the basis for species delimitation and the concept of phylospecies in *Alternaria* sect. *Alternaria* (Fig. 1). All of our isolates grouped together with representative taxa of *A. alternata* and the AASC, and no other species of *Alternaria* sect. *Alternaria,* such as *A. gossypina* and *A. iridiaustralis,* were represented. Most of our isolates grouped with *A. alternata* lineages. The strongly supported (B-PP = 1, ML-BS = 65) AASC clade comprised the 26 isolates (representing the second group of grapevine-derived isolates) together with well-characterized AASC strains. In the seven-locus phylogeny of our isolates from grapevine and reference sequences^23^, we observed a similar arrangement of our isolates (Supplementary Fig. S2). Most of these grouped together with *A. alternata* strains, and 26 showed strong affiliation with the well-supported (B-PP = 0.99, ML-BS = 99) clade of AASC strains. The phylogenetic reconstructions from both the five-locus and seven-locus analyses produced better resolution among the isolates but resulted in similar topologies to the single-locus analysis, with separation of the studied isolates from the other nine phylogenetic species of *Alternaria* sect. *Alternaria*.

### Single-locus trees

Although the single-locus phylogenies produced slight differences in species resolution in the case of the 11 species or species complexes in *Alternaria* sect. *Alternaria*^23^, most species could be distinguished consistently within these phylogenies (Supplementary Fig. S3). Based on the single-locus and multilocus trees, we could also assume that none of our isolates belonged to any of the nine clades representing the species *A. alstroemeriae, A. betae-kenyensis, A. eichhorniae, A. gaisen, A. gossypina, A. iridiaustralis, A. jacinthicola, A. longipes,* and *A. tomato* (Supplementary Fig. S3). Phylogeny based on *rpb2* revealed higher variability among the isolates than analyses using ITS and revealed two distinct groups of the isolates, similarly to the multilocus tree. Analysis of 157 sequences of *Alt a 1*, 144 of *endoPG,* and 170 of OPA 10–2 resulted in assignment of the same two groups. The most appropriate locus for distinguishing AASC isolates from *A. alternata* isolates was OPA 10–2, as AASC sequences were separated (BS = 84) in the single-locus phylogeny of this region. Using *rpb2* also resulted in a distinct clade with (BS = 73). In our analyses, none of the other loci revealed clearly separated clades for all reference sequences of the AASC (Supplementary Fig. S3).

### Untargeted chemical profiling and metabolite identification

**Figure 2:**
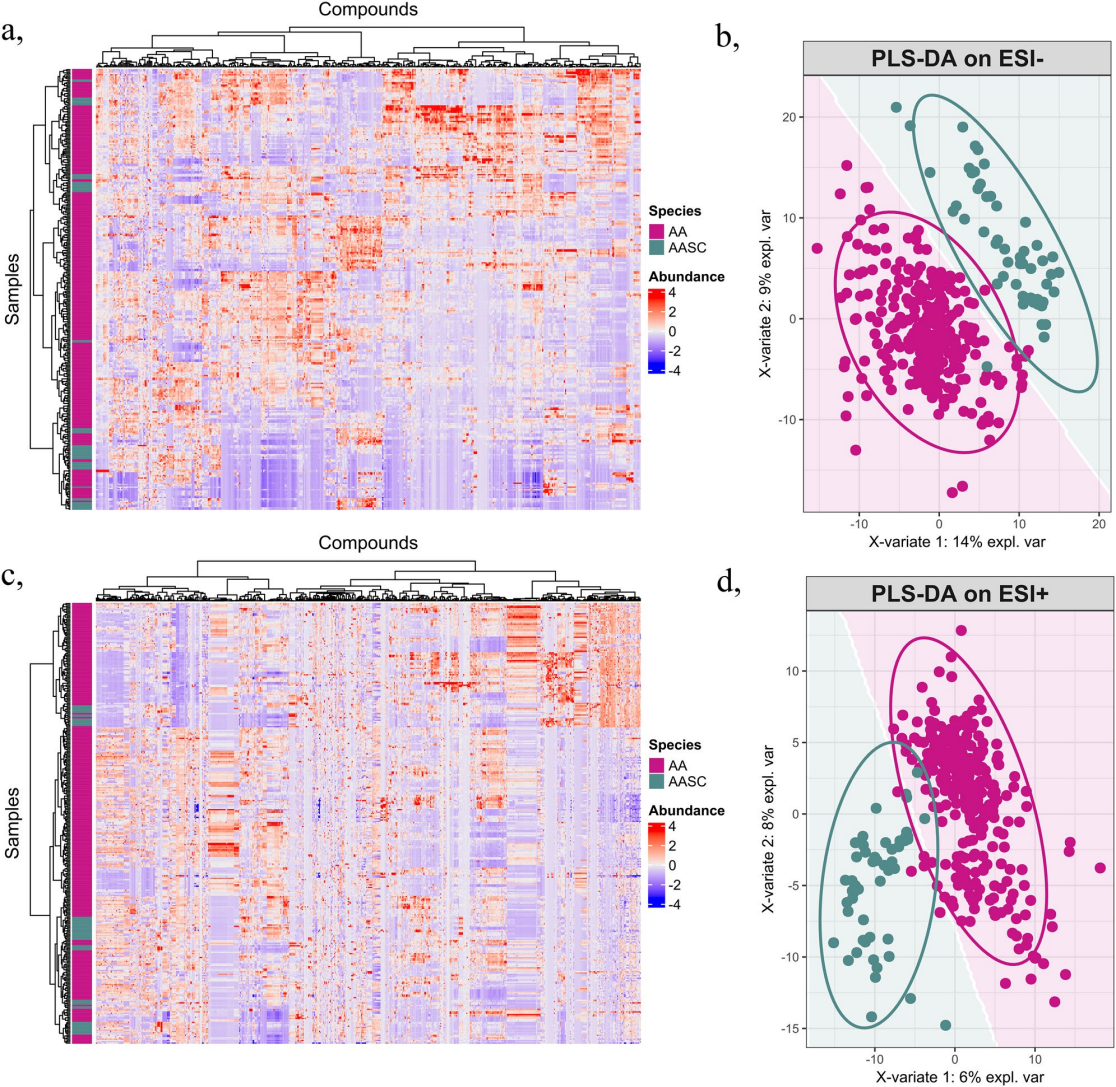
Heatmaps of negative (**a**) and positive (**c**) normalized feature areas across samples. On both columns and rows a hierarchical clustering was performed by Ward’s method with Canberra distances. PLS-DA analysis results of negative and positive datasets are shown on panels (**b**) and (**d**), respectively.

All 170 isolates of *A. alternata* and the AASC from grapevine were analyzed by HPLC coupled to a DAD and high-resolution tandem mass spectrometry (HR-MS/MS) detections for metabolic profiling. Untargeted MS data processing revealed the presence of 647 and 453 distinct molecular features using positive and negative ionization, respectively. PCA of these datasets showed no significant separation of *A. alternata* and the AASC, indicating that the major source of variation is not attributable to the grouping of A. alternata species and the AASC, as shown in the heatmaps (Fig. 2a,c). However, a PLS-DA model built with two components successfully discriminated between the two groups by chemical profile, suggesting a difference in the chemical composition of these groups (Fig. 2b,d). Univariate statistical analysis of each feature as a binary classifier (Supplementary Fig. 9) revealed that 75 and 54 metabolites in positive and negative mode, respectively, could discriminate between *A. alternata* and the AASC. Most of the discriminant metabolites appear to be more abundant in *A. alternata*, with the exception of three features with higher concentrations in the AASC.

**Figure 3:**
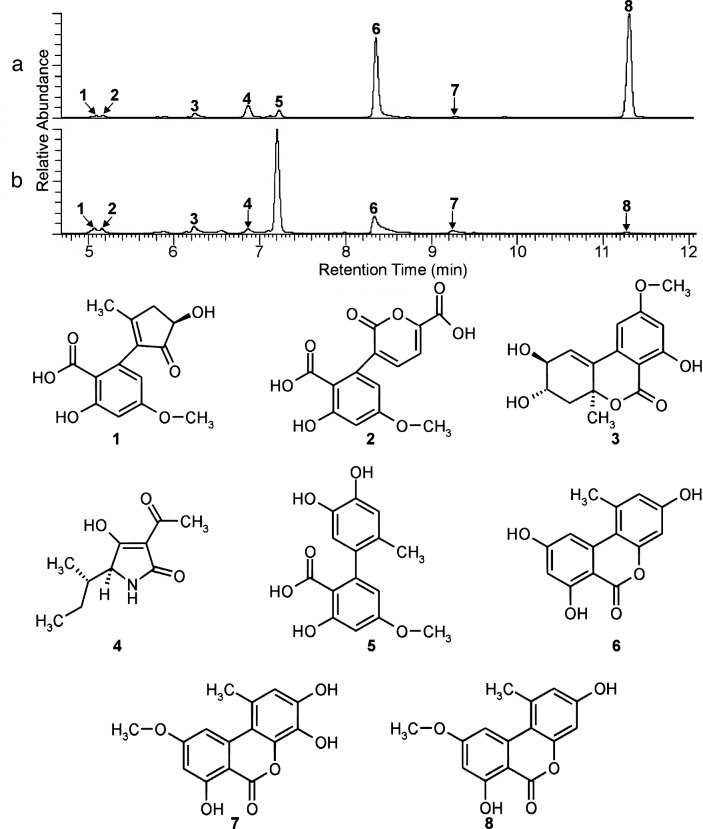
High-performance liquid chromatography ultraviolet spectrophotometry (HPLC–UV) separations (**a**, **b**) of the extracts prepared from *Alternaria alternata* isolate vvmerl3ml5 (**a**) and *A. arborescens* species complex (AASC) isolate vvunid6yl6 (**b**), and the chemical structures of their main compounds **1** alternarienonic acid, **2** alternerian acid, **3** altenuene, **4** L-tenuazonic acid, **5** altenusin, **6** alternariol, **7** 4-hydroxyalternariol methyl ether and **8** alternariol monomethyl ether (λ = 250–600 nm).

The culture extracts contained eight *Alternaria*-specific compounds (Fig. 3), which could be identified using molecular formulas (Table 1) and UV spectra identical to those of known metabolites of *Alternaria* species. We detected alternarienonic acid, alternarian acid, altenuene, *L*-tenuazonic acid, altenusin, alternariol, 4-hydroxyalternariol methyl ether, and alternariol monomethyl ether (Supplementary Figs. S4–S7). All 170 isolates produced these main compounds, although at different concentrations. Although the average concentrations and variances of these metabolites were similar in *A. alternata* and the AASC (negative Z-prime and AUROC < 0.8), two of these, namely altenuene and *L*-tenuazonic acid, differed significantly (Bonferroni-corrected *p* < 0.007) between the two groups, and differences between certain clades were also present. Among these molecules, we observed a distinct pattern for *L*-tenuazonic acid (Supplementary Fig. 8).

**Table 1:** Mass spectrometry (MS) data of compounds **1**–**8**, determined by high-performance liquid chromatography (HPLC) high-resolution Orbitrap-MS and MS/M.

No.^a^	Compound name	Formula	MS mode^b^	Detected ion	Measured mass^c^ (*m/z*)	Calculated mass (*m/z*)	Mass error (ppm)	Characteristic fragment ions^d^, indicated by mass-to-charge ratios (*m/z*) and ion structures^e^
1	Alternarienonic acid	C_14_H_14_O_6_	+	[M + H]^+^	279.0861	279.0863	− 0.912	261.0756 [M + H − H_2_O]^+^; 243.065 [M + H − 2H_2_O]^+^; 233.0807 [M + H − HCOOH]^+^
				[M + Na]^+^	301.0679	301.0683	− 1.193	
			−	[M − H]^−^	277.0719	277.0707	4.567	233.0817 [M − H − CO_2_] −
2	Alternarin acid	C_15_H_12_O_8_	+	[M + H]^+^	321.0603	321.0605	− 0.697	303.0497 [M + H − H_2_O]^+^; 257.0441 [M + H − H_2_O − HCOOH]^+^; 231.0651 [M + H − CO_2_ − HCOOH]^+^; 203.0701 [M + H − CO_2_ − HCOOH − CO]^+^
				[M + NH_4_]^+^	338.0865	338.0870	− 1.458	
			−	[M − H]^−^	319.0462	319.0448	4.251	247.0611 [M − H − CO_2_ − CO]^−^; 231.0660 [M − H − 2CO_2_]^−^; 203.0707 [M − H − 2CO_2_ − CO]^−^
3	Altenuene	C_15_H_16_O_6_	+	[M + H]^+^	293.1015	293.1020	− 1.483	275.0910 [M + H − H_2_O]^+^; 257.0807 [M + H − 2H_2_O]^+^
			−	[M − H]^−^	291.0877	291.0863	4.897	247.0974 [M − H − CO_2_]^−^
				[M + COOH] −	337.0933	337.0918	4.438	
4	L-Tenuazonic acid	C_10_H_15_NO_3_	+	[M + H]^+^	198.1122	198.1125	− 1.615	181.0858 [M + H − NH_3_]^+^; 153.0909 [M + H − NH_3_ − CO]^+^
			−	[M − H]^−^	196.0972	196.0968	1.939	139.0264 [M − H − C_4_H_9_]^−^; 112 [M − H − C_4_H_9_ − HCN]^−^
5	Altenusin	C_15_H_14_O_6_	+	[M + H]^+^	291.0860	291.0863	− 1.184	273.0755[M + H − H_2_O]^+^; 255.0649 [M + H − 2H_2_O]^+^; 245.0807 [M + H − HCOOH]^+^; 227.0701 [M + H − HCOOH − H_2_O]^+^
			−	[M − H]^−^	289.0720	289.0707	4.689	245.0819 [M − H − CO_2_]^−^; 230.0581 [M − H − CO_2_ − CH_3_]^−^
6	Alternariol	C_14_H_10_O_5_	+	[M + H]^+^	259.0598	259.0601	− 1.119	244.0372 [M + H − CH_3_]^+^; 241.0483 [M + H − H_2_O]^+^; 217.0495 [M + H − CH_2_CO]^+^
			−	[M − H]^−^	257.0457	257.0444	4.669	229.0493 [M − H − CO]^−^; 213.0550 [M − H − CO_2_]^−^
7	4-Hydroxyalternariol methyl ether	C_15_H_12_O_6_	+	[M + H]^+^	289.0703	289.0707	− 1.227	271.0592 [M + H − O]^+^; 243.0644 [M + H − CO_2_]^+^
			−	[M − H]^−^	287.0564	287.0550	4.826	272.0328 [M − H − CH_3_]^−^
8	Alternariol methyl ether	C_15_H_12_O_5_	+	[M + H]^+^	273.0752	273.0757	− 1.904	258.0515 [M + H − CH_3_]^+^; 255.0648 [M + H − H_2_O]^+^; 231.0661[M + H − CH_2_CO]^+^
			−	[M − H]^−^	271.0614	271.0601	4.796	256.0377 [M − H − CH_3_]^−^; 228.0435 [M − H − CH_3_ − CO]^−^

^a^Numbers of compounds correspond to those in Fig. 3.

^b^Mass spectrometry (MS) detection was operated in positive (+) and negative (−) ionization modes.

^c,d^High resolution mass spectra and fragment ion spectra can be found in the Supplementary Material (Figs. S2–S4).

^e^Differences between the measured and calculated masses (m/z) of each ion were less than 5.0 ppm.

## Discussion

Based on economic value and hectares cultivated, grapevine is one of the major crops worldwide^56^ and has been described as a host plant of highly diversified microbial communities by many authors in the recent decade. Using both culture-dependent and culture-independent methods, it has been shown that species of *Alternaria* sect*. Alternaria* and related *Alternaria* species are represented among the endophytic fungal communities inhabiting the healthy tissues of grapevine^1–11^. Through the combination of morphological characterization, multigene sequence analysis, and metabolite profiling, this taxonomically challenging genus has undergone several revisions^15–25,29–36,57^ in the decades since its first description^58^. A comprehensive morphological approach by Simmons^57^ resulted in the description of more than 270 *Alternaria* species and the division of the genus into subgeneric sections. *Alternaria* is currently divided into 27 sections, from which members of *Alternaria* sect. *Alternaria* have been identified in significant numbers in our previous work^10^ based on sequences of ITS and *rpb2*. In this study, we subjected endophytic *Alternaria* strains isolated from asymptomatic grapevine leaves and clusters to a combined analysis of five additional genomic loci and metabolite profiling for improved species resolution within the *Alternaria* section.

For multilocus analysis of *Alternaria* isolates, ITS, *rpb2*, *Alt a 1*, *endoPG*, OPA10-2, and KOG1058 were sequenced, the genomic sequences of these loci, along with *gapdh* and *tef1*, have been used previously to evaluate phylogenetic relationships among species in *Alternaria* sect. *Alternaria*^23^. Based on both single-locus and multilocus phylogeny, the *Alternaria* strains obtained from grapevine represented two distinct lineages within *Alternaria* sect. *Alternaria*, considered as *A*. *alternata* and the AASC. Despite taxonomic differences between *A. alternata* and the AASC, their known host range, biology, and growing characteristics are similar. This condition has been confirmed by the fact that many of our isolates belonging to *A. alternata* and the AASC originated from the same plant sample (e.g., vvchar5yl1 and vvchar5yl3, Supplementary Table 2). Our results are consistent with those reported by Lorenzini and Zapparoli^59^ and Tao et al.^60^, who found that grapevine-derived *Alternaria* isolates clearly divided into two major clusters: *A. alternata* and the AASC.

By combining RAPD-PCR, morphological characterization, and metabolite profiling, Polizzotto^12^ identified endophytic *Alternaria* strains originating from grapevine shoots as members of the AASC and the *A. tenuissima* species group; they were distinct from strains belonging to *A. alternata*. This outcome suggests that small-spored *Alternaria* species other than *A. alternata* and the AASC may also be present in grapevine; however, we only detected these two groups in our study. Tao et al.^60^ have described a new species (*A. viniferae*) isolated from pedicels and rachis and closely related to *A. longipes*, which is the other grapevine-associated *Alternaria* strain clustering with *A. alternata* and the AASC based on sequence data from the *gpd* and *Alt a 1* genes. Phylogenetic analysis of ITS sequences clustered all *Alternaria* isolates obtained from withered grapes within a monophyletic clade, while intergenic spacer region (IGS)-RFLP profiles were congruent with those of *A. alternata* and the AASC, as reported by Lorenzini and Zapparoli^59^.

Based on the analysis of ITS, LSU, *gapdh*, TEF, and *rpb2* sequences, Dissanayake et al.^6^ identified all *Alternaria* isolates from grapevine stems as *A*. *alternata *sensu stricto. These results support our findings that not all genomic loci that have been successfully applied in studies of other fungal genera provide sufficient resolution among species in *Alternaria* sect. *Alternaria* but that the combination of informative loci in multigene phylogeny could assist with the more precise delimitation of species.

*Alternaria* isolates from symptomatic pomegranate fruits have also been identified as *A. alternata* and the AASC based on ITS, *tef1*, *gapdh,* and OPA10-2 sequences. Despite the morphological and genetic differences within these fungi, all tested isolates of both *A. alternata* and the AASC had a similar effect and induced similar symptoms of heart rot in pomegranate fruit^61^. By combining *Alt a 1*, *gapdh*, *tef1,* and *tub* gene sequences, Somma et al.^62^ also successfully distinguished *A*. *alternata* from AASC strains isolated from wheat kernels and found *Alt a 1* to be the most informative locus according to the percentage of polymorphic sites. The two species were also found together by Fontaine^63^, who performed multilocus sequence analysis targeting the *endoPG*, *Alt a* 1, and OPA10-2 regions among *Alternaria* isolates from leaves and fruits of apple samples affected by Alternaria leaf blotch (ALB) and Alternaria fruit spot (AFS). These two *Alternaria* taxa are the major cause of ALB and AFS^64^ but colonize grapevine tissues without any visible symptoms.

The presence of *Alternaria* species in agronomic plants is frequently connected with the accumulation of secondary metabolites representing a wide variety of biological activities. The prevalence of these species is of high importance in food safety and plant pathology, as many of the produced metabolites have been classified as mycotoxins or phytotoxins^34^. In recent years, several substrates, such as cereals, fruits, and derived products intended for human or animal consumption, have been analyzed for the presence of *Alternaria* metabolites, focusing mainly on mycotoxins^27^. However, toxicity and modes of action have not been elucidated in detail for all substances. Eight known fungal secondary metabolites were identified in the grapevine-associated *Alternaria* isolates that we investigated, namely alternarienonic acid, alternarian acid, altenuene, *L*-tenuazonic acid, altenusin, alternariol, 4-hydroxyalternariol methyl ether, and alternariol methyl ether (Fig. 3), of which altenuene, *L*-tenuazonic acid, altenusin, alternariol, and alternariol methyl ether have previously been reported in *Alternaria* isolated from different grapevine organs^65–68^. Alternarienonic acid was identified first in cultures of endophytic *Alternaria* spp. isolated from *Polygonum senegalense*^69^ and later from the mangrove plant *Sonneratia alba*^70^ as well as blueberries, walnuts, tomatoes, and wheat^34^. Alternarian acid was identified in cultured *A. mali* isolated from naturally infected tobacco^71^. Toxicity of the dibenzopyrone derivative altenuene was first reported against different bacteria^72^. Several *Alternaria* species also produce various phytotoxins that are both host-specific and non-host-specific. Tenuazonic acid belongs to the tetramic acid derivative group of *Alternaria* metabolites and has been widely detected in several agronomic plants, such as cottonseed and bolls^73^. It has been described as a non-host-specific, nitrogen-containing phytotoxin inhibiting photophosphorylation^74^ and was found to have antimicrobial properties against *Mycobacterium tuberculosis*^75^. Tenuazonic acid has also been linked to several adverse effects on animal species such as mice, chickens, and dogs^76,77^ and has been found to inhibit protein biosynthesis in rat liver cells and Ehrlich ascites by suppressing the release of nascent proteins from the ribosome^78^. It has been detected along with alternariol and alternariol monomethyl ether in food products such as ice wines^79^, cornflakes^80^, sunflower flour^81^, and tomato puree^82^. Tenuazonic acid is also produced by the fungi *Pyricularia oryzae* and *Phoma sorghina*^83,84^. Altenusin is a biphenyl derivative with antioxidant activity and the capability to inhibit various enzymes of *Paracoccidioides brasiliensis* and *Schizosaccharomyces pombe*^85^. Altenusin isolated from the plant *Trixis vauthieri* has been found to act as a potential chemotherapeutic agent to treat leishmaniasis and trypanosomiasis by inhibiting the drug target trypanothione reductase^86^. Altenuene, alternariol, alternariol methyl ether, and 4-hydroxyalternariol methyl ether are dibenzo-α-pyrones possessing antibacterial and antioxidant effects. Like tenuazonic acid, alternariol is a non-host-specific phytotoxin^28^. Furthermore, alternariol induces lipid peroxidation in the epithelium of the human fetal esophagus in vitro^87^ and has been reported to exhibit genotoxic activity by inducing DNA strand breaks in cultured mammalian cells^88–90^. Alternariol methyl ether can also inhibit type II DNA topoisomerases in human colon adenocarcinoma cells^91^. Alternariol is secreted by other fungi in addition to *Alternaria* species, including *Stagonospora nodorum*^92^ and *Phomopsis* sp.^93^. The compound 4-hydroxyalternariol methyl ether and alternariol from endophytic *Alternaria* sp. obtained from the medicinal plant *Salvia miltiorrhiza* have shown antibacterial activities against six pathogenic bacteria^94^. Of note, 4-hydroxyalternariol methyl ether is produced not only by *Alternaria* species but also by endolichenic fungal strains of *Nigrospora* sp.^95^.

The multivariate analyses of the untargeted metabolite characterization we performed found no clear separation among *Alternaria* species in the metabolomic space. However, a PLS-DA model was able to successfully discriminate between the metabolic data from isolates belonging to the AASC and the remaining *A. alternata* isolates. By conducting univariate analysis based on the discriminant ability of the metabolites, we also identified several features with large and significant variation between *A. alternata* and the AASC. Although well-known *Alternaria* metabolites were detected in all isolates at similar concentrations, the amounts of two chemicals, altenuene and *L*-tenuazonic acid, differed significantly between *A. alternata* and the AASC.

The distinct grouping of the AASC among *A. alternata,* revealed previously and supported by our present molecular phylogenetic analyses, is supported by the data obtained from the untargeted metabolic study. This separation of groups may suggest functional differences, which could explain how isolates of the two groups could be found even in the same small tissue samples. Further studies are needed to understand the role of this metabolic and potentially functional diversity in the functioning of the plant microbiome.

### Supplementary Information

Supplementary Figure 1.

Supplementary Figure 2.

Supplementary Figure 3.

Supplementary Figure 4.

Supplementary Figure 5.

Supplementary Figure 6.

Supplementary Figure 7.

Supplementary Figure 8.

Supplementary Figure 9.

Supplementary Legends.

Supplementary Table 1.

Supplementary Table 2.

Supplementary Table 3.

## Data Availability

The datasets used and/or analysed during the current study are available from the corresponding author on reasonable request. DNA sequences of *A. alternata* and *A. arborescens* species complex used in this study were deposited in GenBank (OQ931049–OQ931220, OQ973480–OQ974171).
