# Author Correction: Untargeted metabolomic analyses support the main phylogenetic groups of the common plant-associated *Alternaria* fungi isolated from grapevine (*Vitis vinifera*)

**DOI:** 10.1038/s41598-024-69306-6

**Published:** 2024-08-09

**Authors:** Anna Molnár, Dániel G. Knapp, Miklós Lovas, Gergő Tóth, Imre Boldizsár, Kálmán Zoltán Váczy, Gábor M. Kovács

**Affiliations:** 1 Centre for Research and Development, Eszterházy Károly Catholic University, Leányka utca 6, Eger, 3300 Hungary; 2 Department of Plant Anatomy, Institute of Biology, Eötvös Loránd University, Pázmány Péter sétány 1/C, Budapest, 1117 Hungary; 3 Hevesy György PhD School of Chemistry, Institute of Chemistry, ELTE Eötvös Loránd University, 1117 Budapest, Hungary; 4 Department of Pharmaceutical Chemistry, Semmelweis University, Hőgyes Endre U. 9, Budapest, 1092 Hungary; 5 Department of Pharmacognosy, Semmelweis University, Üllői út 26, Budapest, 1085 Hungary; 6 Plant Protection Institute, Centre for Agricultural Research, Budapest, 1525 Hungary; 7 Present Address: Department of Forestry and Wood Technology, Linnaeus University, Växjö, Sweden

Correction to: *Scientific Reports* 10.1038/s41598-023-46020-3, published online 07 November 2023

The original version of this Article omitted an affiliation for Miklós Lovas. The correct affiliations are listed below.

Centre for Research and Development, Eszterházy Károly Catholic University, Leányka utca 6, Eger 3300, Hungary.

Hevesy György PhD School of Chemistry, Institute of Chemistry, ELTE Eötvös Loránd University, 1117 Budapest, Hungary.

The original Article has been corrected.

