# Supplementary figures and images for: Untargeted metabolomic analyses support the main phylogenetic groups of the common plant-associated Alternaria fungi isolated from grapevine (Vitis vinifera)
Source: Sci Rep. 2023 Nov 7;13:19298. doi: 10.1038/s41598-023-46020-3 (PMC10630412; doi:10.1038/s41598-023-46020-3)

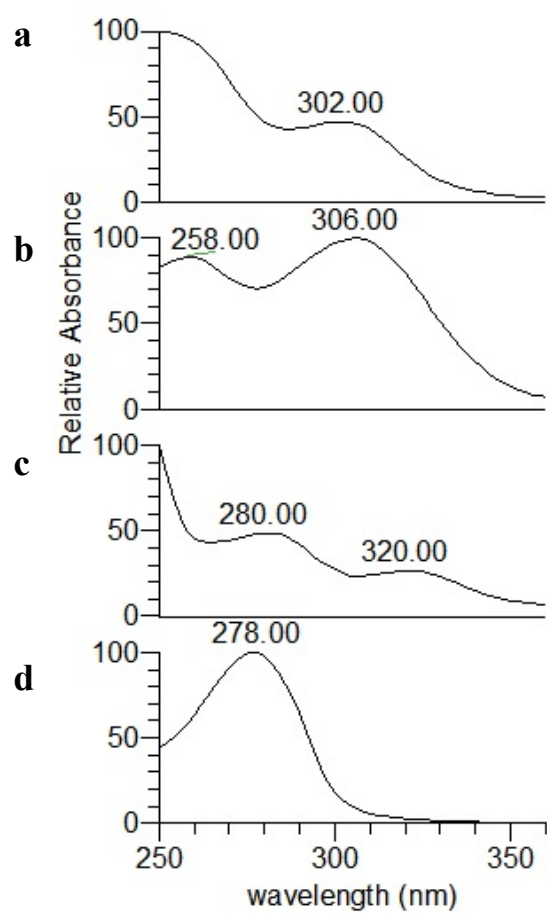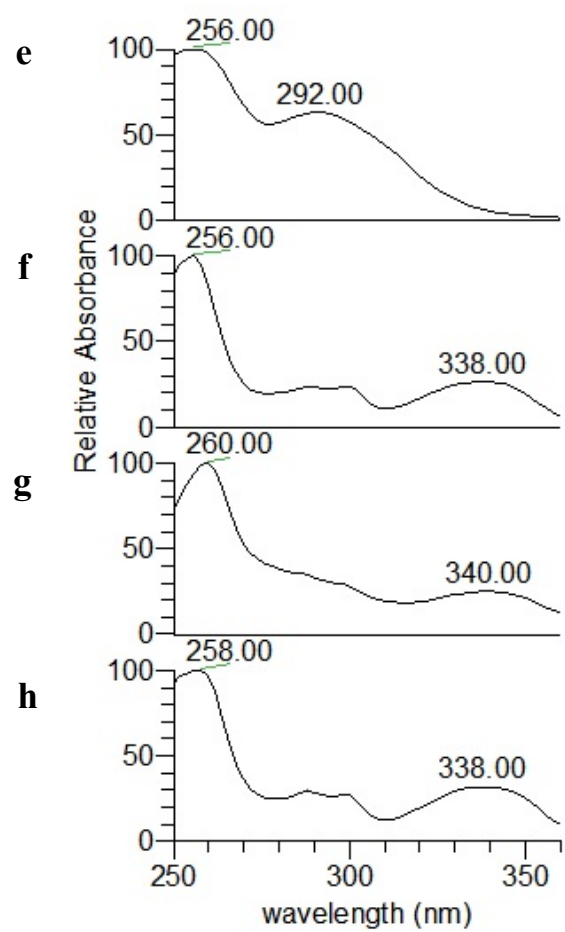

Supplement: Supplementary file 4 — Supplementary Figure 4. [file 41598_2023_46020_MOESM4_ESM.pdf]

**a**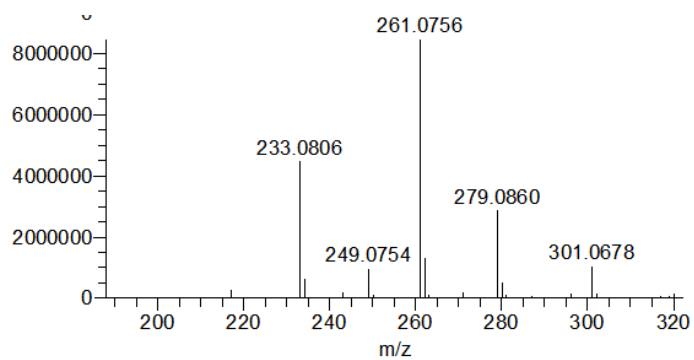**b**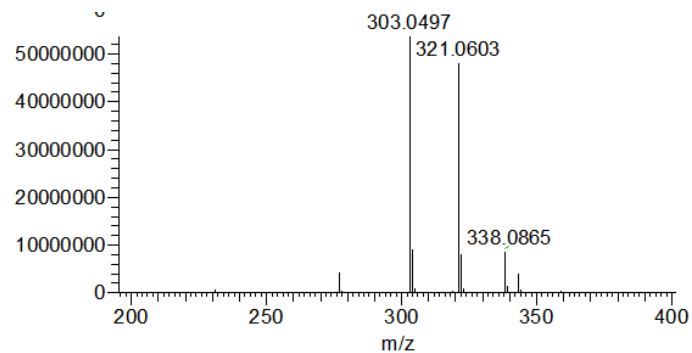**c**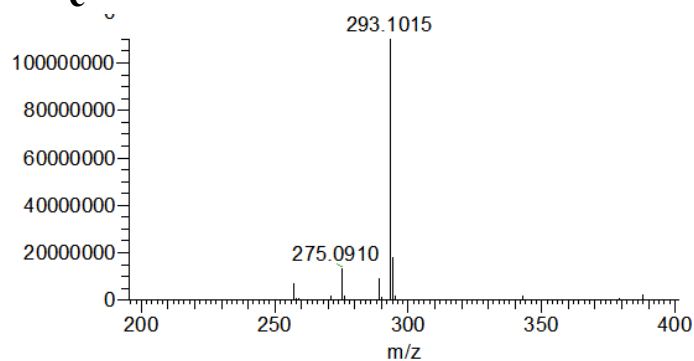**d**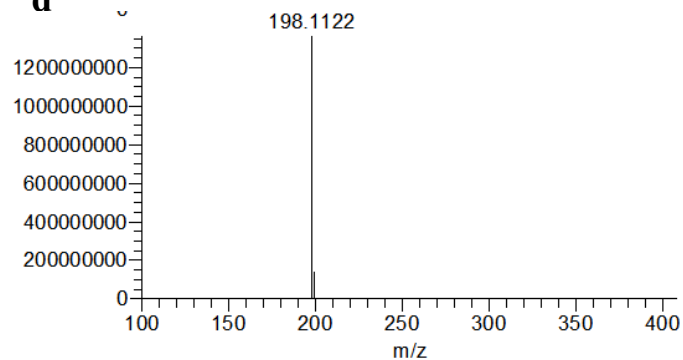**e**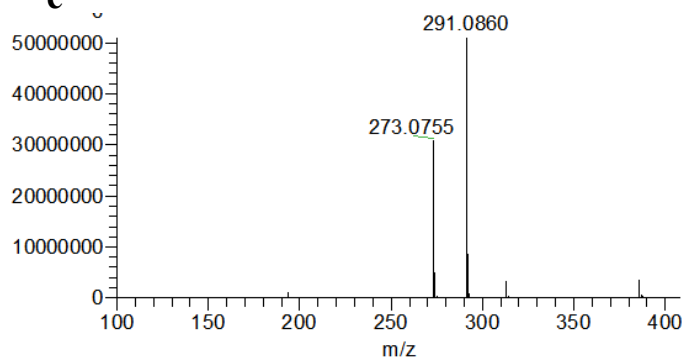**f**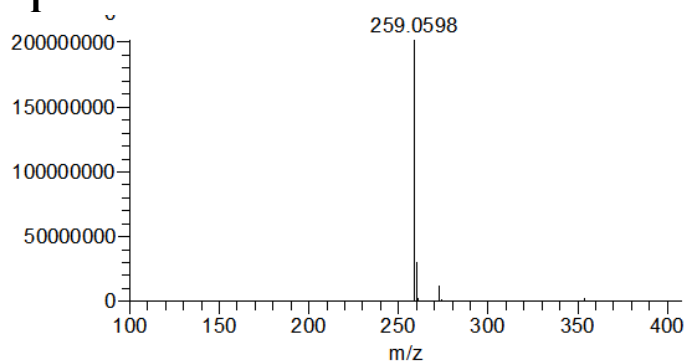**g**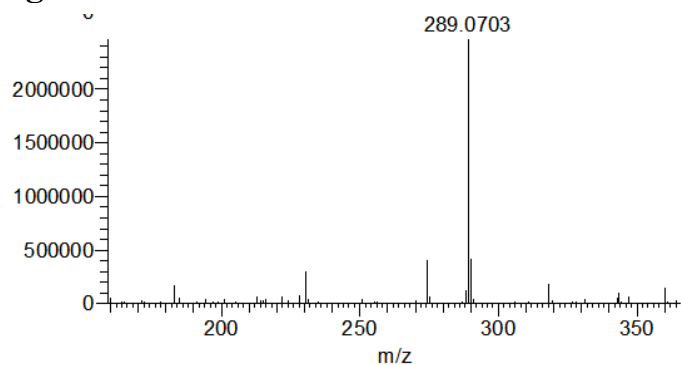**h**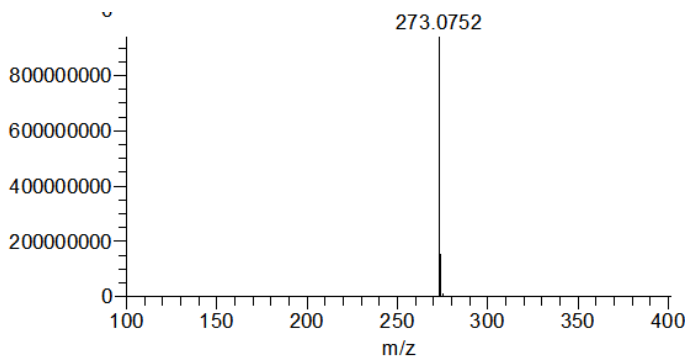

Supplement: Supplementary file 5 — Supplementary Figure 5. [file 41598_2023_46020_MOESM5_ESM.pdf]

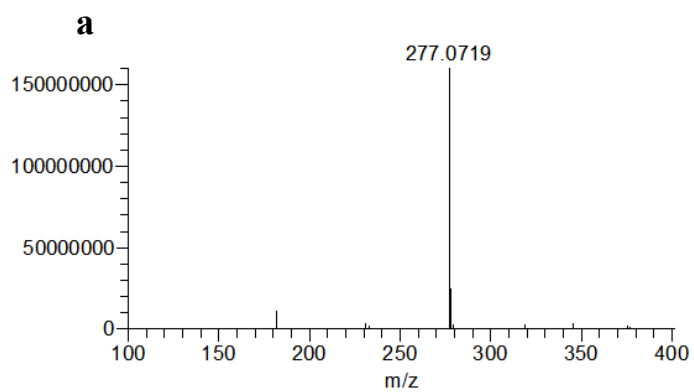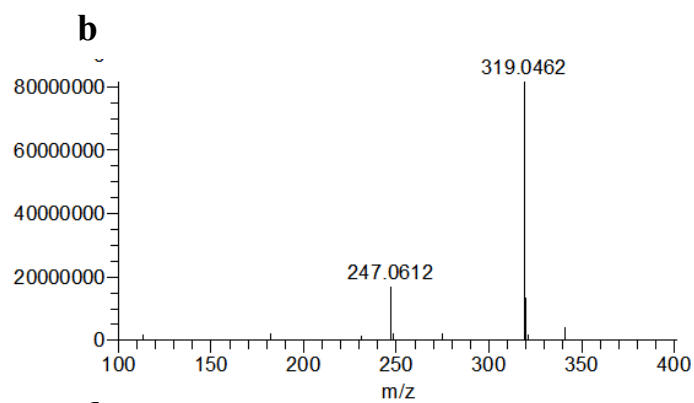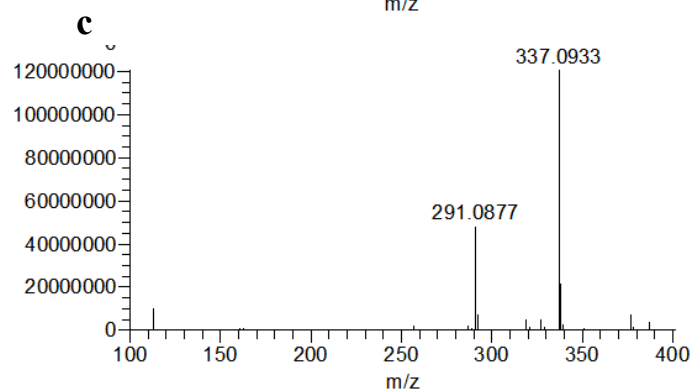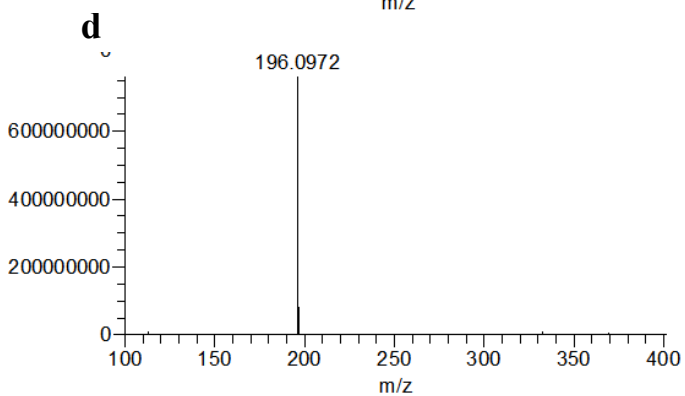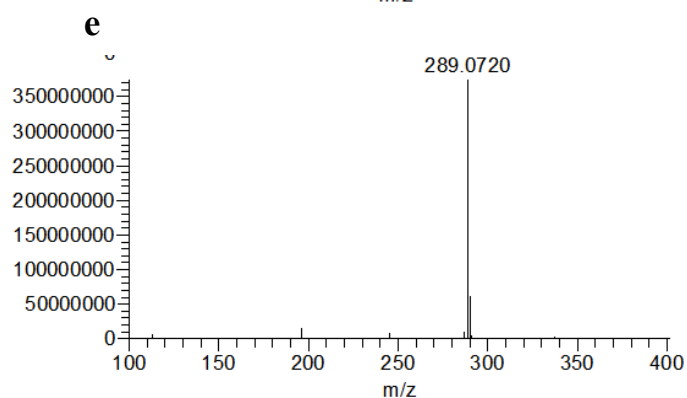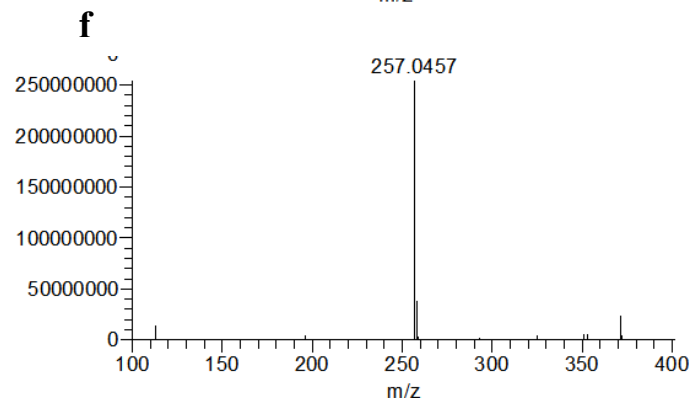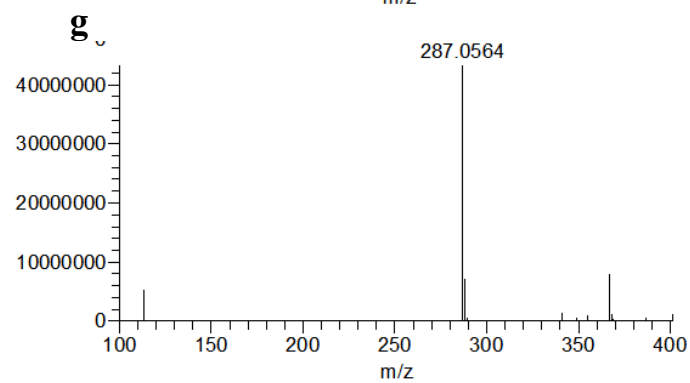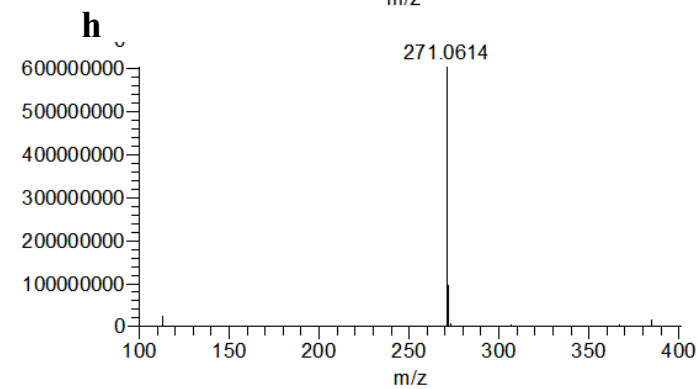

Supplement: Supplementary file 6 — Supplementary Figure 6. [file 41598_2023_46020_MOESM6_ESM.pdf]

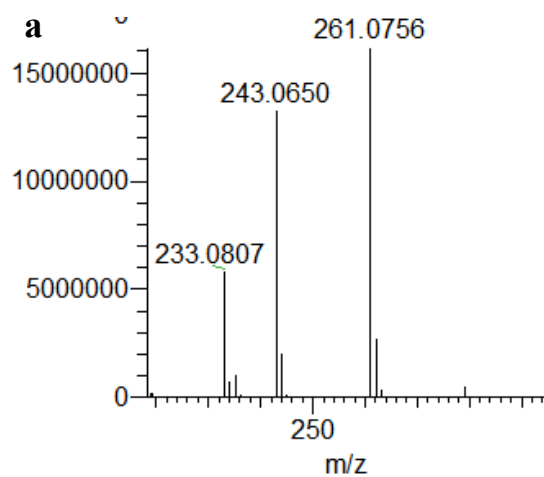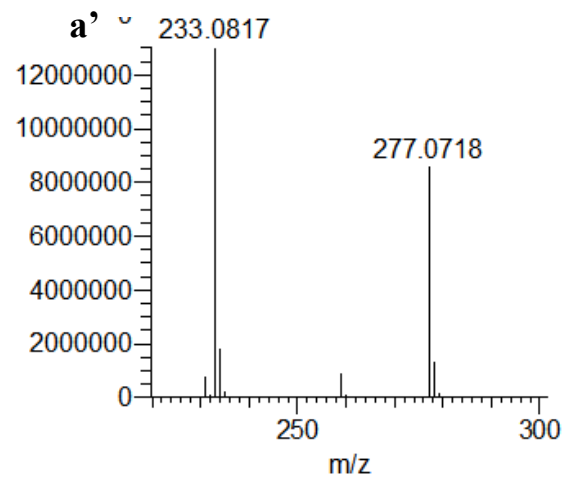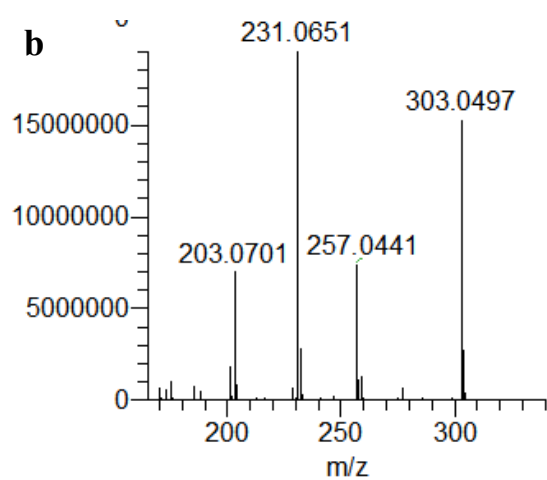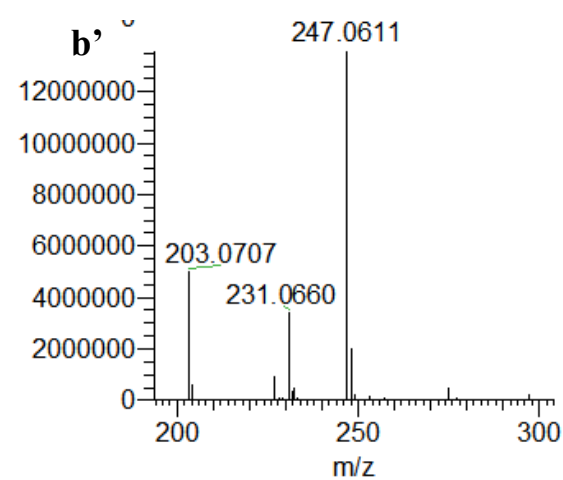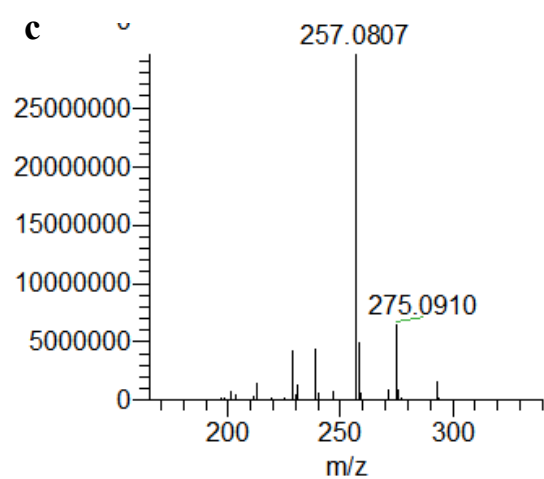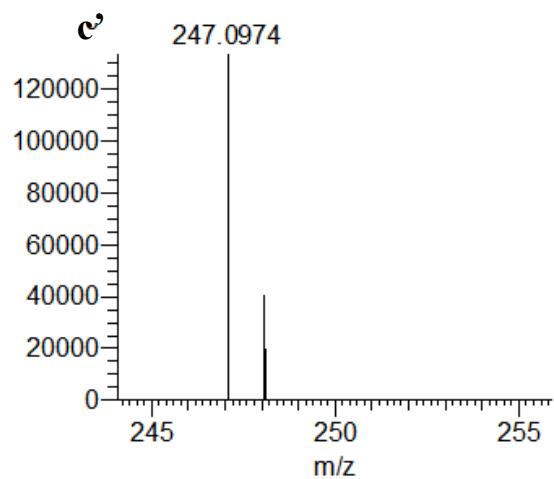

**d**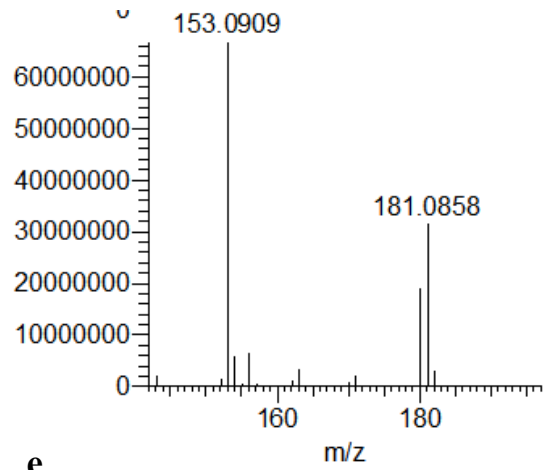**d'**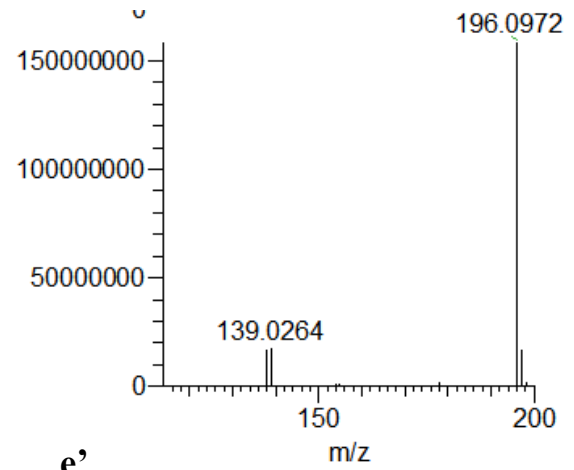**e**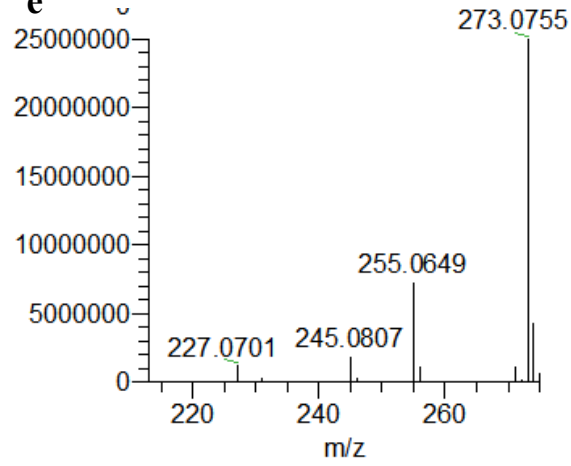**e'**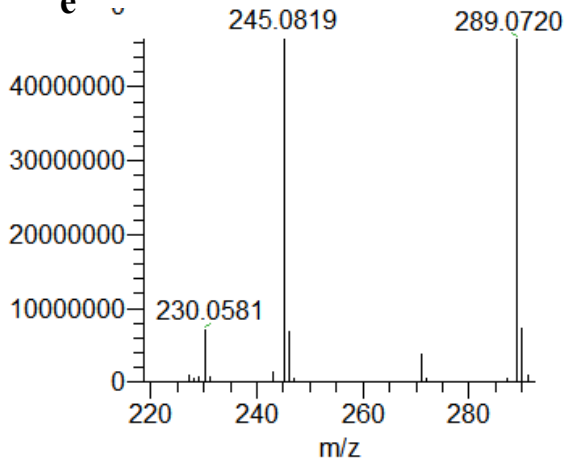**f**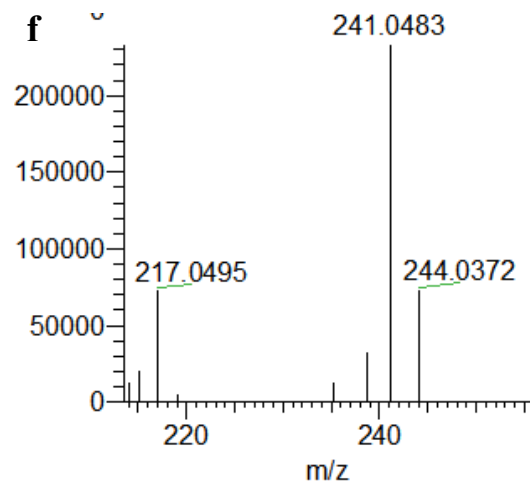**f'**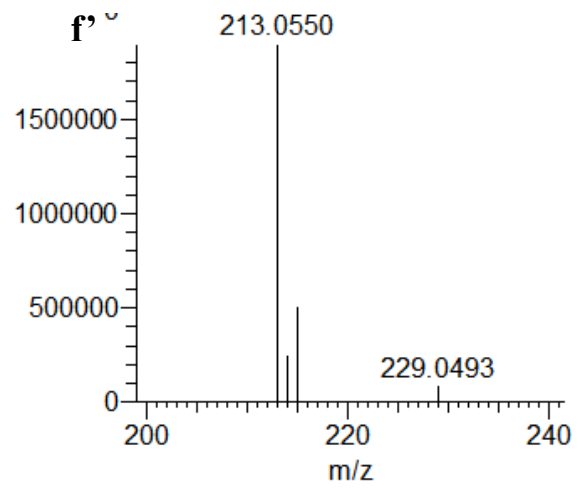

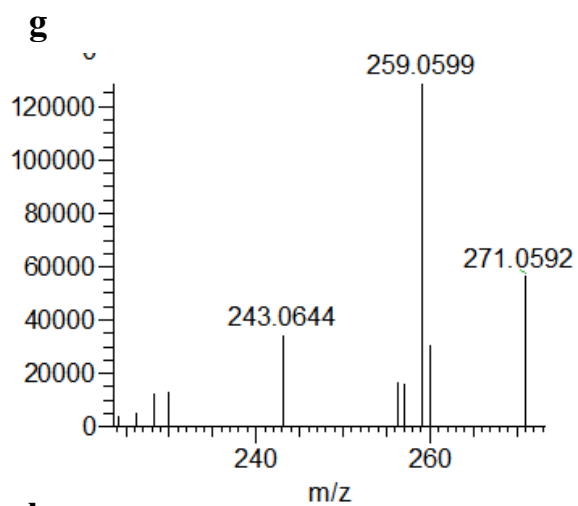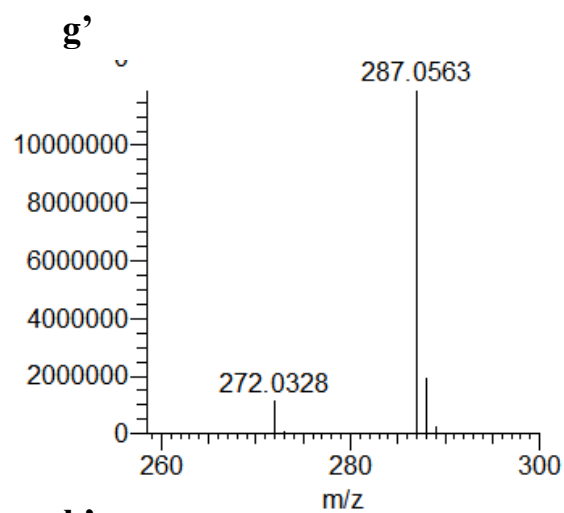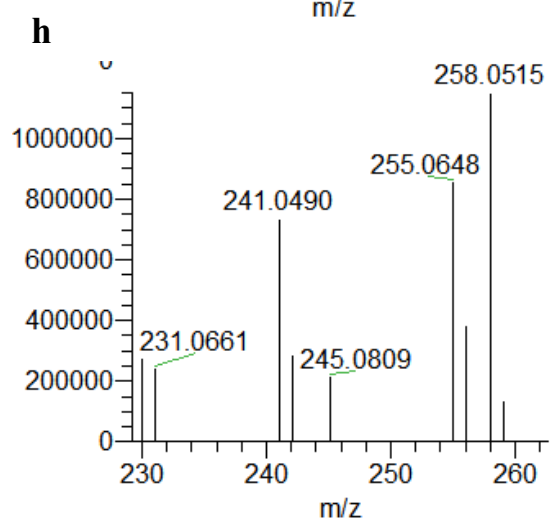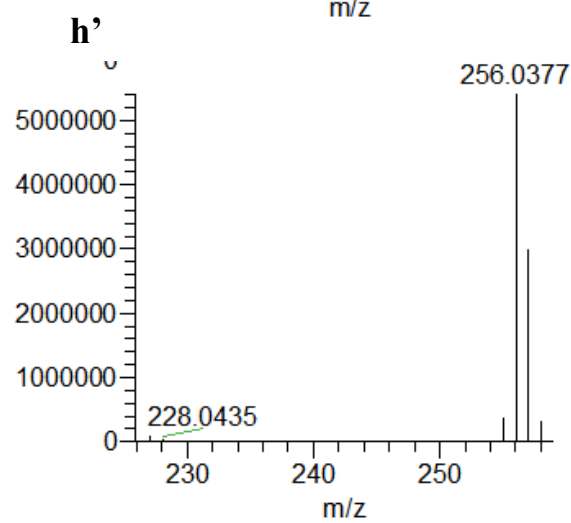

Supplement: Supplementary file 7 — Supplementary Figure 7. [file 41598_2023_46020_MOESM7_ESM.pdf]
