# Supplementary material for: Untargeted metabolomic analyses support the main phylogenetic groups of the common plant-associated Alternaria fungi isolated from grapevine (Vitis vinifera)
Source: Sci Rep. 2023 Nov 7;13:19298. doi: 10.1038/s41598-023-46020-3 (PMC10630412; doi:10.1038/s41598-023-46020-3)
Supplement: Supplementary file 10 — Supplementary Legends. [file 41598_2023_46020_MOESM10_ESM.docx]

**Untargeted metabolomic analyses support the main phylogenetic groups of the common plant-associated *Alternaria* fungi isolated from grapevine (*Vitis vinifera*)**

Anna Molnár, Dániel G. Knapp, Miklós Lovas, Gergő Tóth, Imre Boldizsár, Kálmán Zoltán Váczy, Gábor M. Kovács

**Supplementary Material**

**Supplementary Figure 1.** Phylogenetic tree of all *Alternaria* isolates collected from grapevine leaves and clusters. The 50% majority rule consensus phylogram inferred from Bayesian analysis of the combined dataset of six loci (rpb2, ITS, Alt a 1, endoPG, OPA10-2, KOG1058). Bayesian posterior probabilities (≥ 0.90) are shown before slashes, ML bootstrap support (≥ 70) is shown after slashes.

**Supplementary Figure 2.** Phylogenetic tree of all *Alternaria* isolates collected from grapevine leaves and clusters and reference *Alternaria* strains *sensu* Woudenberg et al. [23]. The 50% majority rule consensus phylogram inferred from Bayesian analysis of the combined dataset of seven loci (*rpb2*, ITS, *Alt a 1*, *endoPG*, OPA10-2, *tef1*, *gapdh*). Bayesian posterior probabilities (≥ 0.90) are shown before slashes, ML bootstrap support (≥ 70) is shown after slashes. Isolates representing the lineages of *A. alternata* (violet) and *A. arborescens* species complex (AASC, dark green) in this study are shown in bold. Sequences were rooted to *A. alternantherae, A. perpunctulata, A. solani, A. porri, A. tagetica, A. macrospora, A. pseudorostrata* and *A. dauci*. Scale bar indicates 0.2 expected changes per site.

**Supplementary Figure 3.** Single-locus phylogenetic trees of all *Alternaria* isolates collected from grapevine leaves and clusters. The 50% majority rule consensus phylogram inferred from Maximum likelihood analysis of the seven loci, ITS, *rpb2*, *Alt a 1*, *endoPG*, OPA10-2, *tef1* and *gapdh*. ML bootstrap support values (≥ 70) are shown above or below the internal branches.

**Supplementary Figure 4.** UV spectra of compounds **1** alternarienonic acid (a), **2** alternerian acid (b), **3** altenuene (c), **4** L-tenuazonic acid (d), altenusin **5** (e), alternariol **6** (f), 4-hydroxyalternariol methyl ether **7** (g) and alternariol monomethyl ether **8** (h).

**Supplementary Figure 5.** High resolution mass spectra of compounds **1** alternarienonic acid (A), **2** alternerian acid (B), **3** altenuene (C), **4** L-tenuazonic acid (D), altenusin **5** (E), alternariol **6** (F), 4-hydroxyalternariol methyl ether **7** (G) and alternariol monomethyl ether **8** (H), obtained by high-resolution mass spectrometry operated in positive ionization mode.

**Supplementary Figure 6.** High resolution mass spectra of compounds **1** alternarienonic acid (a), **2**  alternerian acid (b), **3** altenuene (c), **4** L-tenuazonic acid (d), altenusin **5** (e), alternariol **6** (f), 4-hydroxyalternariol methyl ether **7** (g) and alternariol monomethyl ether **8** (h), obtained by high-resolution mass spectrometry operated in negative ionization mode.

**Supplementary Figure 7.** High resolution mass fragment ion spectra of compounds **1** alternarienonic acid (a, a’), **2** alternerian acid (b, b’), **3** altenuene (c, c’), **4** L-tenuazonic acid (d, d’), altenusin **5** (e, e’), alternariol **6** (f, f’), 4-hydroxyalternariol methyl ether **7** (g, g’) and alternariol monomethyl ether **8** (h, h’), obtained by fragmentation of mass isolation widths that cover the m/z (mass-to-charge ratio) values of selected compounds, using positive ionization mode (spectra a–h) and negative ionization mode (spectra a’–h’) and collision induced dissociation energies (CID) of 15 eV, 30 eV and 45 eV).

**Supplementary Figure 8.** Boxplots of the 8 identified *Alternaria*-specific metabolites across clades. Abundances are visualized as normalized area values.

**Supplementary Figure 9.** Heatmaps illustrating positive (a, b, c) and negative (d, e, f) discriminant ions in all samples, selected based on their AUROC, fold-change, and p-values. A Volcano plot (b, c) demonstrates selected metabolites with appropriate fold-change and satisfactory p-values. AUROC plots for positive (c) and negative (f) features assess their discriminant ability between *A. alternata* and AASC. In the top-left corner of the plots, features with significantly different means between groups and high AUROC values (>0.8) indicate robust discriminant ability. The color of each dot in figures b, c, e, and f indicates higher abundance in AA or AASC, while dot size correlates with the Z-prime factor of the feature.

**Supplementary Table 1.** Isolates used in this study with reference isolates *sensu* Woudenberg et al. [23].

**Supplementary Table 2.** Details of *Alternaria* isolates collected from leaves and clusters of different grapevine cultivars.

**Supplementary Table 3.** Primer sequence and optimal annealing temperature of each locus evaluated in this study.
