# Supplementary material for: Untargeted metabolomic analyses support the main phylogenetic groups of the common plant-associated Alternaria fungi isolated from grapevine (Vitis vinifera)
Source: Sci Rep. 2023 Nov 7;13:19298. doi: 10.1038/s41598-023-46020-3 (PMC10630412; doi:10.1038/s41598-023-46020-3)
Supplement: Supplementary file 11 — Supplementary Table 1. [file 41598_2023_46020_MOESM11_ESM.docx]

| Supplementary Table 1. Isolates used in this study with reference isolates *sensu* Woudenberg et al. [23]. | | | | | | | | | |
| --- | --- | --- | --- | --- | --- | --- | --- | --- | --- |
| **Species, strain number** | **Origin** | **GenBank accession numbers** | | | | | | | |
|  |  | ITS | RPB2 | ALTA1 | endoPG | OPA10-2 | KOG1058 | TEF1 | GAPDH |
| ***Alternaria alstroemeriae*** |  |  |  |  |  |  |  |  |  |
| CBS 118808 | USA, *Alstroemeria* sp. | KP124296 | KP124764 | KP123845 | KP123993 | KP124601 |  | KP125071 | KP124153 |
| CBS 118809 | Australia, *Alstroemeria* sp. | KP124297 | KP124765 | - | KP123994 | KP124602 |  | KP125072 | KP124154 |
| ***Alternaria alternantherae*** |  |  |  |  |  |  |  |  |  |
| CBS 124392 | China, *Solanum melongena* | KC584179 | KC584374 | KP123846 | - | - |  | KC584633 | KC584096 |
| ***Alternaria alternata*** |  |  |  |  |  |  |  |  |  |
| CBS 102.47 | USA, *Citrus sinensis* | KP124304 | KP124773 | KP123855 | KP124002 | KP124610 |  | KP125080 | KP124161 |
| CBS 102595 | USA, *Citrus jambhiri* | FJ266476 | KC584408 | AY563306 | KP124029 | KP124636 |  | KC584666 | AY562411 |
| CBS 102596 | USA, Citrus jambhir | KP124328 | KP124796 | KP123877 | KP124030 | KP124637 |  | KP125104 | KP124183 |
| CBS 102598 | USA*, Minneola tangelo* | KP124329 | KP124797 | KP12387 | KP124031 | KP124638 |  | KP125105 | KP124184 |
| CBS 102599 | Turkey*, Minneola tangelo* | KP124330 | KP124798 | KP123879 | KP124032 | KP124639 |  | KP125106 | KP124185 |
| CBS 102600 | USA, *Citrus reticulata* | KP124331 | KP124799 | KP123880 | KP124033 | KP124640 |  | KP125107 | KP124186 |
| CBS 102602 | Turkey, *Minneola tangelo* | KP124332 | KP124800 | KP123881 | AY295023 | KP124641 |  | KP125108 | KP124187 |
| CBS 102603 | Israel, *Minneola tangelo* | KP124333 | KP124801 | KP123882 | KP124034 | KP124642 |  | KP125109 | KP124188 |
| CBS 102604 | Israel, *Minneola tangelo* | KP124334 | KP124802 | AY563305 | KP124035 | KP124643 |  | KP125110 | AY562410 |
| CBS 103.33 | Egypt, soil | KP124302 | KP124770 | KP123852 | KP123999 | KP124607 |  | KP125077 | KP124159 |
| CBS 104.26 | Unknown, unknown | KP124299 | KP124767 | KP123848 | KP123995 | KP124603 |  | KP125074 | KP124156 |
| CBS 106. 24 | USA, *Malus sylvestris* | KP124298 | KP124766 | KP123847 | AY295020 | JQ800620 |  | KP125073 | KP124155 |
| CBS 106.34 | Unknown, *Linum usitatissimum* | Y17071 | KP124771 | KP123853 | KP123853 | KP124608 |  | KP125078 | JQ646308 |
| CBS 107.27 | USA, *Citrus limonium* | KP124300 | KP124768 | KP123849 | KP123996 | KP124604 |  | KP125075 | KP124157 |
| CBS 107.53 | Japan, *Pyrus pyrifolia* | KP124305 | KP124774 | KP123858 | KP124005 | KP124613 |  | KP125081 | KP124162 |
| CBS 109455 | Canada, human arm tissue | KP124335 | KP124803 | KP123883 | KP124036 | KP124644 |  | KP125111 | KP124189 |
| CBS 109803 | Germany, human skin | KP124336 | KP124804 | KP123884 | KP124037 | KP124645 |  | KP125112 | KP124190 |
| CBS 110027 | Germany, human eye | KP124337 | KP124805 | KP123885 | KP124038 | KP124646 |  | KP125113 | KP124191 |
| CBS 112249 | Unknown, unknown | KP124338 | KP124806 | KP123886 | KP124039 | KP124648 |  | KP125114 | KP124192 |
| CBS 112251 | Unknown, unknown | KP124339 | KP124807 | KP123887 | KP124040 | KP124649 |  | KP125115 | KP124193 |
| CBS 112252 | Unknown, unknown | KP124340 | KP124808 | KP123888 | KP124041 | KP124650 |  | KP125116 | KP124194 |
| CBS 113013 | South Africa, *Malus domestica* | KP124341 | KP125117 | KP124809 | KP123889 | KP124042 |  | KP124651 | KP124195 |
| CBS 113014 | South Africa, *Malus domestica* | KP124342 | KP124810 | KP123890 | KP124043 | KP124652 |  | KP125118 | KP124196 |
| CBS 113015 | South Africa, *Malus domestica* | KP124343 | KP124811 | KP123891 | KP124044 | KP124653 |  | KP125119 | KP124197 |
| Supplementary Table 1. Isolates used in this study with reference isolates *sensu* Woudenberg et al. [23]. | | | | | | | | | |
| **Species, strain number** | **Origin** | **GenBank accession numbers** | | | | | | | |
|  |  | ITS | RPB2 | ALTA1 | endoPG | OPA10-2 | KOG1058 | TEF1 | GAPDH |
| CBS113024 | South Africa, *Minneola tangelo* | KP124344 | KP124812 | KP123892 | KP124045 | KP124654 |  | KP125120 | KP124198 |
| CBS 113025 | South Africa, *Citrus clementina* | KP124345 | KP124813 | KP123893 | KP124046 | KP124655 |  | KP125121 | KP124199 |
| CBS 113054 | South Africa, *Malus domestica* | KP124346 | KP124814 | KP123894 | KP124047 | KP124656 |  | KP125122 | KP124200 |
| CBS 115188 | South Africa, *Citrus clementina* | KP124349 | KP124817 | KP123897 | KP124050 | KP124659 |  | KP125125 | KP124203 |
| CBS 115190 | South Africa, *Citrus sinensis* | KP124350 | KP124818 | KP123898 | KP124051 | KP124660 |  | KP125126 | KP124204 |
| CBS 115199 | South Africa, *Minneola tangelo* | KP124351 | KP124819 | KP123899 | KP124052 | KP124661 |  | KP125127 | KP124205 |
| CBS 115200 | South Africa, *Minneola tangelo* | KP124352 | KP124820 | KP123900 | KP124053 | KP124662 |  | KP125128 | KP124206 |
| CBS 115616 | India, *Arachis hypogaea* | AF347031 | KC584375 | AY563301 | JQ811978 | KP124663 |  | KC584634 | AY278808 |
| CBS 116749 | Netherlands, unknown | KP124353 | KP124821 | KP123901 | KP124054 | KP124664 |  | KP125129 | KP124207 |
| CBS 117.44 | Denmark, *Godetia* sp. | KP124303 | KP124772 | KP123854 | KP124001 | KP124609 |  | KP125079 | KP124160 |
| CBS 117130 | Italy, *Arbutus unedo* | KP124354 | KP124822 | KP123902 | KP124055 | KP124665 |  | KP125130 | KP124208 |
| CBS 117143 | Italy, *Capsicum annuum* | KP124355 | KP124823 | KP123903 | KP124056 | KP124666 |  | KP125131 | KP124209 |
| CBS 118811 | USA, *Brassica oleracea* | KP124356 | KP124824 | KP123904 | KP124057 | KP124667 |  | KP125132 | KP124210 |
| CBS 118812 | USA, *Daucus carota* | KC584193 | KC584393 | KP123905 | KP124058 | KP124668 |  | KC584652 | KC584112 |
| CBS 118814 | USA, *Solanum lycopersicum* | KP124357 | KP124825 | KP123906 | KP124059 | KP124669 |  | KP125133 | KP124211 |
| CBS 118815 | USA, *Solanum lycopersicum* | KP124358 | KP124826 | KP123907 | KP124060 | KP124670 |  | KP125134 | KP124212 |
| CBS 118818 | USA, *Vaccinium* sp. | KP124359 | KP124827 | KP123908 | KP124061 | KP124671 |  | KP125135 | KP124213 |
| CBS 119115 | Greece, *Prunus* sp | KP124360 | KP124828 | KP123909 | KP124062 | - |  | KP125136 | KP124214 |
| CBS 119399 | USA, *Minneola tangelo* | KP124361 | KP124829 | KP123910 | KP124063 | KP124672 |  | KP125137 | JQ646328 |
| CBS 119408 | USA, *Euphorbia esula* | KP124362 | KP124830 | JQ646410 | KP124064 | KP124673 |  | KP125138 | JQ646326 |
| CBS 119543 | USA, *Citrus paradisi* | KP124363 | KP124831 | KP123911 | KP124065 | KP124674 |  | KP125139 | KP124215 |
| CBS 120829 | Greece, *Punica granatum* | KP124364 | KP124832 | KP123912 | KP124066 | KP124675 |  | KP125140 | KP124216 |
| CBS 121336 | USA, *Allium* sp. | KJ862254 | KP124833 | KJ862259 | KP124067 | KP124676 |  | KP125141 | KJ862255 |
| CBS 121344 | Israel, *Minneola tangelo* | KP124365 | KP124834 | KP123913 | KP124068 | KP124677 |  | KP125142 | KP124217 |
| CBS 121346 | South Africa, *Minneola tangelo* | KP124366 | KP124835 | KP123914 | KP124069 | KP124678 |  | KP125143 | KP124218 |
| CBS 121348 | China, *Platycodon grandiflorus* | KP124367 | KP124836 | KP123915 | KP124070 | KP124679 |  | KP125144 | KP124219 |
| CBS 121454 | USA, *Cuscuta gronovii* | AF278836 | KP124837 | JQ646402 | KP124071 | KP124680 |  | KP125145 | AY278812 |
| CBS 121455 | China, *Broussonetia papyrifera* | KP124368 | KP124838 | KP123916 | KP124072 | KP124681 |  | KP125146 | KP124220 |
| Supplementary Table 1. Isolates used in this study with reference isolates *sensu* Woudenberg et al. [23]. | | | | | | | | | |
| **Species, strain number** | **Origin** | **GenBank accession numbers** | | | | | | | |
|  |  | ITS | RPB2 | ALTA1 | endoPG | OPA10-2 | KOG1058 | TEF1 | GAPDH |
| CBS 121456 | China, *Sanguisorba officinalis* | KP124369 | KP124839 | KP123917 | KP124073 | KP124682 |  | KP125147 | KP124221 |
| CBS 121492 | China, *Cucumis melo* | KP124370 | KP124840 | KP123918 | KP124074 | KP124683 |  | KP125148 | KP124222 |
| CBS 121544 | USA, *Cucumis sativus* | KP124371 | KP124841 | KP123919 | KP124075 | KP124684 |  | KP125149 | KP124223 |
| CBS 121547 | China, *Pyrus bretschneideri* | KP124372 | KP124842 | KP123920 | KP124076 | KP124685 |  | KP125150 | KP124224 |
| CBS 124277 | Denmark, *Prunus* sp | KP124373 | KP124843 | KP123921 | KP124077 | KP124686 |  | KP125151 | KP124225 |
| CBS 124278 | Denmark, *Prunus* sp. | KP124374 | KP124844 | KP123922 | KP124078 | KP124687 |  | KP125152 | KP124226 |
| CBS 125606 | India, human | KP124375 | KP124845 | KP123923 | KP124079 | KP124688 |  | KP125153 | KP124227 |
| CBS 126071 | Namibia, soil | KP124376 | KP124846 | KP123924 | KP124080 | KP124689 |  | KP125154 | KP124228 |
| CBS 126072 | Namibia, soil | KP124377 | KP124847 | KP123925 | KP124081 | KP124690 |  | KP125155 | KP124229 |
| CBS 126908 | USA, soil | KP124378 | KP124848 | KP123926 | KP124082 | KP124691 |  | KP125156 | KP124230 |
| CBS 126910 | USA, soil | KP124379 | KP124849 | KP123927 | KP124083 | KP124692 |  | KP125157 | KP124231 |
| CBS 127334 | USA, soil | KP124380 | KP124850 | KP123928 | KP124084 | KP124693 |  | KP125158 | KP124232 |
| CBS 127671 | USA, *Stanleya pinnata* | KP124381 | KP124851 | KP123929 | KP124085 | KP124694 |  | KP125159 | KP124233 |
| CBS 127672 | USA, *Astragalus bisulcatus* | KP124382 | KP124852 | KP123930 | KP124086 | KP124695 |  | KP125160 | KP124234 |
| CBS 130254 | India, human sputum | KP124383 | KP124853 | KP123931 | KP124087 | KP124696 |  | KP125161 | KP124235 |
| CBS 130255 | India, human sputum | KP124384 | KP124854 | KP123932 | KP124088 | KP124697 |  | KP125162 | KP124236 |
| CBS 130258 | India, human sputum | KP124385 | KP124855 | KP123933 | KP124089 | KP124698 |  | KP125163 | KP124237 |
| CBS 130259 | India, human sputum | KP124386 | KP124856 | KP123934 | KP124090 | KP124699 |  | KP125164 | KP124238 |
| CBS 130260 | India, human sputum | KP124387 | KP124857 | KP123935 | KP124091 | KP124700 |  | KP125165 | KP124239 |
| CBS 130261 | India, human sputum | KP124388 | KP124858 | KP123936 | KP124092 | KP124701 |  | KP125166 | KP124240 |
| CBS 130262 | India, human sputum | KP124389 | KP124859 | KP123937 | KP124093 | KP124702 |  | KP125167 | KP124241 |
| CBS 130263 | India, human sputum | KP124389 | KP124859 | KP123937 | KP124093 | KP124702 |  | KP125167 | KP124241 |
| CBS 130265 | India, human sputum | KP124391 | KP124861 | KP123939 | KP124095 | KP124704 |  | KP125169 | KP124243 |
| CBS 154.31 | USA, *Staphylea trifolia* | KP124301 | KP124769 | KP123851 | KP123998 | KP124606 |  | KP125076 | KP124158 |
| CBS 174.52 | USA, *Anemone occidentalis* | KC584228 | DQ677964 | KP123856 | KP123856 | KP124611 |  | KC584704 | KC584152 |
| CBS 175.52 | USA, *Juncus mertensianus* | KC584227 | KC584445 | KP123857 | KP124004 | KP124612 |  | KC584703 | KC584151 |
| CBS 175.80 | Italy, unknown | KP124313 | KP124781 | KP123866 | KP124013 | KP124620 |  | KP125089 | JQ646324 |
| CBS 192.81 | Egypt, *Citrus sinensis* | KP124314 | KP124782 | KP123867 | KP124014 | KP124621 |  | KP125090 | KP124170 |
| Supplementary Table 1. Isolates used in this study with reference isolates *sensu* Woudenberg et al. [23]. | | | | | | | | | |
| **Species, strain number** | **Origin** | **GenBank accession numbers** | | | | | | | |
|  |  | ITS | RPB2 | ALTA1 | endoPG | OPA10-2 | KOG1058 | TEF1 | GAPDH |
| CBS 194.86 | USA, *Quercus* sp. | KP124316 | KP124784 | KP123869 | KP124016 | KP124623 |  | KP125092 | KP124172 |
| CBS 195.86 | Canada, *Euphorbia esula* | KP124317 | KP124785 | JQ646398 | KP124017 | KP124624 |  | KP125093 | KP124173 |
| CBS 198.74 | Kuwait, soil | KP124310 | - | KP123863 | KP124010 | KP124617 |  | KP125086 | KP124167 |
| CBS 267.77 | USA, *Citrus paradisi* | KP124311 | KP124779 | KP123864 | KP124011 | KP124618 |  | KP125087 | KP124168 |
| CBS 447.86 | Marocco*, Malva* sp. | KP124318 | KP124786 | JQ646397 | KP124018 | KP124625 |  | KP125094 | JQ646314 |
| CBS 479.90 | Japan, *Citrus unshiu* | KP124319 | KP124787 | KP123870 | KP124019 | KP124626 |  | KP125095 | KP124174 |
| CBS 595.93 | Japan, *Pyrus pyrifolia* | KP124320 | KP124788 | JQ646399 | KP124020 | KP124627 |  | KP125096 | KP124175 |
| CBS 603.78 | USA, air | KP124312 | KP124780 | KP123865 | KP124012 | KP124619 |  | KP125088 | KP124169 |
| CBS 612.72 | Germany, *Senecio cineraria* | KP124308 | KP124777 | KP123861 | KP124008 | KP124615 |  | KP125084 | KP124165 |
| CBS 620.83 | USA, *Nicotiana tabacum* | KP124315 | KP124783 | KP123868 | KP124015 | KP124622 |  | KP125091 | KP124171 |
| CBS 639.97 | Greece, *Helianthus annuus* | KP124327 | KP124795 | KP123876 | KP124028 | KP124635 |  | KP125103 | KP124182 |
| CBS 686.68 | Sahara, desert sand | KP124306 | KP124775 | KP123859 | KP124006 | KP124614 |  | KP125082 | KP124163 |
| CBS 795.72 | USA, *Plantago aristida* | KP124309 | KP124778 | KP123862 | KP124009 | KP124616 |  | KP125085 | KP124166 |
| CBS 806.96 | Papua New Guinea, *Cyperaceae* | KP124325 | KP124793 | KP123874 | KP124025 | KP124631 |  | KP125101 | KP124180 |
| CBS 826.68 | Germany, *Lolium* sp. | KP124307 | KP124776 | KP123860 | KP124007 | - |  | KP125083 | KP124164 |
| CBS 877.95 | India, human, sinusitis | KP124321 | KP124789 | KP123871 | KP124021 | - |  | KP125097 | KP124176 |
| CBS 880.95 | Belgium, *Fragaria vesca* | KP124322 | KP124790 | - | KP124022 | KP124628 |  | KP125098 | KP124177 |
| CBS 911.97 | India, *Artemisia brevifolia* | KP124326 | KP124794 | KP123875 | KP124027 | KP124634 |  | KP125102 | KP124181 |
| CBS 916.96 | India, *Arachis hypogaea* | AF347031 | KC584375 | AY563301 | JQ811978 | KP124632 |  | KC584634 | AY278808 |
| CBS 918.96 | UK, *Dianthus chinensis* | AF347032 | KC584435 | AY563302 | KP124026 | KP124633 |  | KC584693 | AY278809 |
| CBS 965.95 | India, *Triticum* sp. | KP124323 | KP124791 | KP123872 | KP124023 | KP124629 |  | KP125099 | KP124178 |
| CBS 966.95 | India, *Solanum lycopersicum* | KP124324 | KP124792 | KP123873 | KP124024 | KP124630 |  | KP125100 | KP124179 |
| vvchar5b1 | Hungary, *Vitis vinifera* | OQ931195 | OQ974088 | OQ973497 | OQ973662 | OQ973838 | OQ973785 |  |  |
| vvchar5b11 | Hungary, *Vitis vinifera* | OQ931138 | OQ974089 | OQ973499 | OQ973664 | OQ973839 | - |  |  |
| vvchar5b12 | Hungary, *Vitis vinifera* | OQ931139 | OQ974090 | OQ973500 | OQ973665 | OQ973867 | OQ973786 |  |  |
| vvchar5b13 | Hungary, *Vitis vinifera* | OQ931195 | OQ974146 | OQ973501 | OQ973750 | OQ973868 | - |  |  |
| vvchar5b2 | Hungary, *Vitis vinifera* | OQ931139 | OQ974091 | OQ973498 | OQ973663 | OQ973866 | - |  |  |
| vvchar5rp1 | Hungary, *Vitis vinifera* | OQ931054 | OQ974005 | OQ973508 | OQ973639 | OQ973844 | OQ973788 |  |  |
| Supplementary Table 1. Isolates used in this study with reference isolates *sensu* Woudenberg et al. [23]. | | | | | | | | | |
| **Species, strain number** | **Origin** | **GenBank accession numbers** | | | | | | | |
|  |  | ITS | RPB2 | ALTA1 | endoPG | OPA10-2 | KOG1058 | TEF1 | GAPDH |
| vvchar5rp2 | Hungary, *Vitis vinifera* | OQ931091 | OQ974043 | OQ973509 | - | OQ973845 | - |  |  |
| vvchar5rp8 | Hungary, *Vitis vinifera* | OQ931092 | OQ974044 | OQ973511 | - | OQ973846 | - |  |  |
| vvchar5yl16 | Hungary, *Vitis vinifera* | OQ931089 | OQ974041 | - | - | OQ973841 | - |  |  |
| vvchar5yl17 | Hungary, *Vitis vinifera* | OQ931053 | OQ974004 | OQ973507 | OQ973638 | OQ973842 | - |  |  |
| vvchar5yl3 | Hungary, *Vitis vinifera* | OQ931090 | OQ974042 | OQ973504 | - | OQ973840 | - |  |  |
| vvfurm1ml2 | Hungary, *Vitis vinifera* | OQ931147 | OQ974098 | OQ973481 | OQ973653 | OQ973856 | - |  |  |
| vvfurm1yl1 | Hungary, *Vitis vinifera* | OQ931049 | OQ974000 | OQ973480 | OQ973636 | OQ973830 | OQ973780 |  |  |
| vvkada9ml2 | Hungary, *Vitis vinifera* | OQ931205 | OQ974156 | - | OQ973769 | OQ973908 | - |  |  |
| vvkada9ml4 | Hungary, *Vitis vinifera* | OQ931104 | OQ974056 | OQ973554 | - | OQ973909 | - |  |  |
| vvkada9ml5 | Hungary, *Vitis vinifera* | OQ931152 | OQ974103 | OQ973555 | OQ973679 | OQ973910 | OQ973807 |  |  |
| vvkada9ml7 | Hungary, *Vitis vinifera* | OQ931206 | OQ974157 | OQ973556 | OQ973770 | OQ973911 | - |  |  |
| vvkada9yl11 | Hungary, *Vitis vinifera* | OQ931204 | OQ974155 | OQ973552 | OQ973768 | OQ973906 | - |  |  |
| vvkada9yl12 | Hungary, *Vitis vinifera* | OQ931103 | OQ974055 | OQ973553 | OQ973678 | OQ973907 | - |  |  |
| vvkada9yl4 | Hungary, *Vitis vinifera* | OQ931141 | OQ974092 | OQ973550 | OQ973677 | OQ973904 | OQ973805 |  |  |
| vvkada9yl8 | Hungary, *Vitis vinifera* | OQ931062 | OQ974013 | OQ973551 | OQ973647 | OQ973905 | OQ973806 |  |  |
| vvlean4ml10 | Hungary, *Vitis vinifera* | OQ931052 | OQ974003 | OQ973494 | - | OQ973837 | OQ973784 |  |  |
| vvlean4yl4 | Hungary, *Vitis vinifera* | OQ931194 | OQ974145 | OQ973490 | OQ973749 | OQ973860 | - |  |  |
| vvmerl3b7 | Hungary, *Vitis vinifera* | OQ931086 | OQ974038 | OQ973484 | - | OQ973832 | - |  |  |
| vvmerl3ml5 | Hungary, *Vitis vinifera* | OQ931193 | OQ974144 | OQ973486 | OQ973748 | OQ973858 | OQ973782 |  |  |
| vvmerl3ml10 | Hungary, *Vitis vinifera* | OQ931051 | OQ974002 | OQ973488 | - | OQ973834 | OQ973783 |  |  |
| vvmerl3rp1 | Hungary, *Vitis vinifera* | OQ931087 | OQ974039 | OQ973489 | - | OQ973835 | - |  |  |
| vvmerl3yl3 | Hungary, *Vitis vinifera* | OQ931136 | OQ974087 | OQ973485 | OQ973655 | OQ973833 | - |  |  |
| vvpinn2b7 | Hungary, *Vitis vinifera* | OQ931050 | OQ974001 | OQ973482 | OQ973637 | OQ973831 | - |  |  |
| vvunid10ml1 | Hungary, *Vitis vinifera* | OQ931105 | OQ974057 | OQ973557 | OQ973680 | OQ973912 | OQ973808 |  |  |
| vvunid10ml2 | Hungary, *Vitis vinifera* | OQ931063 | OQ974014 | OQ973558 | OQ973648 | OQ973913 | OQ973809 |  |  |
| vvunid10ml7 | Hungary, *Vitis vinifera* | OQ931142 | OQ974093 | OQ973559 | OQ973681 | OQ973914 | - |  |  |
| vvunid10ml8 | Hungary, *Vitis vinifera* | OQ931106 | OQ974058 | OQ973560 | OQ973682 | OQ973915 | OQ973810 |  |  |
|  |  |  |  |  |  |  |  |  |  |
| Supplementary Table 1. Isolates used in this study with reference isolates *sensu* Woudenberg et al. [23]. | | | | | | | | | |
| **Species, strain number** | **Origin** | **GenBank accession numbers** | | | | | | | |
|  |  | ITS | RPB2 | ALTA1 | endoPG | OPA10-2 | KOG1058 | TEF1 | GAPDH |
| vvunid10rp1 | Hungary, *Vitis vinifera* | OQ931207 | OQ974158 | OQ973561 | OQ973771 | OQ973916 | - |  |  |
| vvunid10rp3 | Hungary, *Vitis vinifera* | OQ931107 | OQ974059 | OQ973562 | OQ973683 | OQ973917 | - |  |  |
| vvunid10rp5 | Hungary, *Vitis vinifera* | OQ931153 | OQ974104 | OQ973563 | OQ973684 | OQ973918 | - |  |  |
| vvunid11b1 | Hungary, *Vitis vinifera* | OQ931154 | OQ974105 | OQ973564 | OQ973685 | OQ973919 | - |  |  |
| vvunid11ml1 | Hungary, *Vitis vinifera* | OQ931208 | OQ974159 | OQ973565 | OQ973772 | OQ973920 | OQ973811 |  |  |
| vvunid11rp10 | Hungary, *Vitis vinifera* | OQ931209 | OQ974160 | OQ973567 | OQ973773 | OQ973922 | - |  |  |
| vvunid11rp11 | Hungary, *Vitis vinifera* | OQ931210 | OQ974161 | - | OQ973774 | OQ973923 | - |  |  |
| vvunid11rp16 | Hungary, *Vitis vinifera* | OQ931108 | OQ974060 | OQ973568 | OQ973687 | OQ973924 | - |  |  |
| vvunid11rp22 | Hungary, *Vitis vinifera* | OQ931109 | OQ974061 | OQ973569 | OQ973688 | OQ973925 | - |  |  |
| vvunid11rp6 | Hungary, *Vitis vinifera* | OQ931155 | OQ974106 | OQ973566 | OQ973686 | OQ973921 | - |  |  |
| vvunid12ml1 | Hungary, *Vitis vinifera* | OQ931112 | OQ974064 | OQ973576 | OQ973692 | OQ973932 | - |  |  |
| vvunid12ml12 | Hungary, *Vitis vinifera* | OQ931156 | OQ974107 | OQ973580 | OQ973695 | OQ973936 | - |  |  |
| vvunid12ml15 | Hungary, *Vitis vinifera* | OQ931064 | OQ974015 | OQ973581 | OQ973649 | OQ973937 | OQ973815 |  |  |
| vvunid12ml16 | Hungary, *Vitis vinifera* | OQ931156 | OQ974016 | OQ973582 | OQ973650 | OQ973938 | - |  |  |
| vvunid12ml17 | Hungary, *Vitis vinifera* | OQ931113 | OQ974065 | OQ973583 | OQ973696 | OQ973939 | - |  |  |
| vvunid12ml2 | Hungary, *Vitis vinifera* | OQ931157 | OQ974108 | OQ973577 | OQ973693 | OQ973933 | - |  |  |
| vvunid12ml8 | Hungary, *Vitis vinifera* | OQ931158 | OQ974109 | OQ973578 | OQ973694 | OQ973934 | OQ973814 |  |  |
| vvunid12ml9 | Hungary, *Vitis vinifera* | OQ931065 | OQ974017 | OQ973579 | - | OQ973935 | - |  |  |
| vvunid12rp1 | Hungary, *Vitis vinifera* | OQ931159 | OQ974110 | - | OQ973697 | OQ973940 | - |  |  |
| vvunid12rp2 | Hungary, *Vitis vinifera* | OQ931114 | OQ974066 | OQ973584 | OQ973698 | OQ973941 | - |  |  |
| vvunid12yl1 | Hungary, *Vitis vinifera* | OQ931211 | OQ974162 | OQ973571 | OQ973776 | OQ973927 | OQ973813 |  |  |
| vvunid12yl10 | Hungary, *Vitis vinifera* | OQ931110 | OQ974062 | OQ973574 | OQ973691 | OQ973931 | - |  |  |
| vvunid12yl2 | Hungary, *Vitis vinifera* | OQ931111 | OQ974063 | OQ973572 | OQ973689 | OQ973928 | - |  |  |
| vvunid12yl3 | Hungary, *Vitis vinifera* | OQ931212 | OQ974163 | OQ973573 | OQ973777 | OQ973929 | - |  |  |
| vvunid12yl6 | Hungary, *Vitis vinifera* | OQ931143 | OQ974094 | OQ973575 | OQ973690 | OQ973930 | - |  |  |
| vvunid13ml1 | Hungary, *Vitis vinifera* | OQ931213 | OQ974164 | - | OQ973778 | OQ973945 | - |  |  |
|  |  |  |  |  |  |  |  |  |  |
|  |  |  |  |  |  |  |  |  |  |
| Supplementary Table 1. Isolates used in this study with reference isolates *sensu* Woudenberg et al. [23]. | | | | | | | | | |
| **Species, strain number** | **Origin** | **GenBank accession numbers** | | | | | | | |
|  |  | ITS | RPB2 | ALTA1 | endoPG | OPA10-2 | KOG1058 | TEF1 | GAPDH |
| vvunid13ml2 | Hungary, *Vitis vinifera* | OQ931116 | - | OQ973586 | OQ973701 | OQ973944 | - |  |  |
| vvunid13rp10 | Hungary, *Vitis vinifera* | OQ931161 | OQ974112 | OQ973589 | OQ973704 | OQ973948 | - |  |  |
| vvunid13rp13 | Hungary, *Vitis vinifera* | OQ931162 | OQ974113 | OQ973590 | OQ973705 | OQ973949 | OQ973817 |  |  |
| vvunid13rp4 | Hungary, *Vitis vinifera* | OQ931163 | OQ974114 | OQ973587 | OQ973702 | OQ973946 | - |  |  |
| vvunid13rp5 | Hungary, *Vitis vinifera* | OQ931164 | OQ974115 | OQ973588 | OQ973703 | OQ973947 | - |  |  |
| vvunid13yl1 | Hungary, *Vitis vinifera* | OQ931115 | OQ974067 | - | OQ973699 | OQ973942 | - |  |  |
| vvunid13yl6 | Hungary, *Vitis vinifera* | OQ931160 | OQ974111 | OQ973585 | OQ973700 | OQ973943 | OQ973816 |  |  |
| vvunid14ml10 | Hungary, *Vitis vinifera* | OQ931066 | OQ974018 | OQ973600 | OQ973651 | OQ973959 | OQ973820 |  |  |
| vvunid14ml11 | Hungary, *Vitis vinifera* | OQ931119 | OQ974070 | OQ973601 | OQ973713 | OQ973960 | - |  |  |
| vvunid14ml12 | Hungary, *Vitis vinifera* | OQ931067 | OQ974019 | OQ973602 | OQ973652 | OQ973961 | OQ973821 |  |  |
| vvunid14ml13 | Hungary, *Vitis vinifera* | OQ931169 | OQ974120 | OQ973603 | OQ973714 | OQ973962 | - |  |  |
| vvunid14ml7 | Hungary, *Vitis vinifera* | OQ931068 | OQ974020 | OQ973599 | OQ973712 | OQ973958 | - |  |  |
| vvunid14yl1 | Hungary, *Vitis vinifera* | OQ931165 | OQ974116 | OQ973591 | OQ973706 | OQ973950 | - |  |  |
| vvunid14yl10 | Hungary, *Vitis vinifera* | OQ931166 | OQ974117 | OQ973595 | OQ973709 | OQ973954 | - |  |  |
| vvunid14yl12 | Hungary, *Vitis vinifera* | OQ931117 | OQ974068 | OQ973597 | OQ973710 | OQ973956 | - |  |  |
| vvunid14yl14 | Hungary, *Vitis vinifera* | OQ931167 | OQ974118 | OQ973598 | OQ973711 | OQ973957 | - |  |  |
| vvunid14yl4 | Hungary, *Vitis vinifera* | OQ931168 | OQ974119 | OQ973592 | OQ973707 | OQ973951 | - |  |  |
| vvunid14yl5 | Hungary, *Vitis vinifera* | OQ931118 | OQ974069 | OQ973593 | OQ973708 | OQ973952 | - |  |  |
| vvunid14yl7 | Hungary, *Vitis vinifera* | OQ931214 | OQ974165 | OQ973594 | - | OQ973953 | OQ973818 |  |  |
| vvunid15b10 | Hungary, *Vitis vinifera* | OQ931120 | OQ974071 | OQ973606 | OQ973717 | OQ973965 | - |  |  |
| vvunid15b2 | Hungary, *Vitis vinifera* | OQ931069 | OQ974021 | OQ973604 | OQ973715 | OQ973963 | - |  |  |
| vvunid15b3 | Hungary, *Vitis vinifera* | OQ931121 | OQ974072 | OQ973605 | OQ973716 | OQ973964 | - |  |  |
| vvunid15ml1 | Hungary, *Vitis vinifera* | OQ931124 | OQ974075 | OQ973613 | OQ973723 | OQ973973 | - |  |  |
| vvunid15ml7 | Hungary, *Vitis vinifera* | OQ931125 | OQ974076 | OQ973614 | OQ973724 | OQ973974 | - |  |  |
| vvunid15rp1 | Hungary, *Vitis vinifera* | OQ931126 | OQ974077 | OQ973615 | OQ973725 | OQ973975 | - |  |  |
| vvunid15rp14 | Hungary, *Vitis vinifera* | OQ931073 | OQ974025 | OQ973617 | OQ973727 | OQ973978 | - |  |  |
| vvunid15rp2 | Hungary, *Vitis vinifera* | OQ931216 | OQ974167 | - | - | OQ973976 | - |  |  |
| vvunid15rp3 | Hungary, *Vitis vinifera* | OQ931074 | OQ974026 | OQ973616 | OQ973726 | OQ973977 | - |  |  |
| Supplementary Table 1. Isolates used in this study with reference isolates *sensu* Woudenberg et al. [23]. | | | | | | | | | |
| **Species, strain number** | **Origin** | **GenBank accession numbers** | | | | | | | |
|  |  | ITS | RPB2 | ALTA1 | endoPG | OPA10-2 | KOG1058 | TEF1 | GAPDH |
| vvunid15yl1 | Hungary, *Vitis vinifera* | OQ931122 | OQ974022 | OQ973607 | OQ973718 | OQ973966 | - |  |  |
| vvunid15yl10 | Hungary, *Vitis vinifera* | OQ931071 | OQ974023 | OQ973611 | OQ973721 | OQ973970 | - |  |  |
| vvunid15yl12 | Hungary, *Vitis vinifera* | OQ931122 | OQ974073 | OQ973612 | OQ973722 | OQ973971 | - |  |  |
| vvunid15yl15 | Hungary, *Vitis vinifera* | OQ931215 | OQ974166 | - | - | OQ973972 | - |  |  |
| vvunid15yl2 | Hungary, *Vitis vinifera* | OQ931072 | OQ974024 | OQ973608 | - | OQ973967 | OQ973822 |  |  |
| vvunid15yl3 | Hungary, *Vitis vinifera* | OQ931123 | OQ974074 | OQ973609 | OQ973719 | OQ973968 | - |  |  |
| vvunid15yl7 | Hungary, *Vitis vinifera* | OQ931144 | OQ974095 | OQ973610 | OQ973720 | OQ973969 | - |  |  |
| vvunid16b1 | Hungary, *Vitis vinifera* | OQ931127 | OQ974078 | OQ973618 | OQ973728 | OQ973979 | - |  |  |
| vvunid16b10 | Hungary, *Vitis vinifera* | OQ931145 | OQ974096 | OQ973619 | OQ973729 | OQ973980 | OQ973823 |  |  |
| vvunid16ml1 | Hungary, *Vitis vinifera* | OQ931218 | OQ974169 | - | - | OQ973990 | - |  |  |
| vvunid16ml2 | Hungary, *Vitis vinifera* | OQ931130 | OQ974081 | OQ973628 | OQ973738 | OQ973991 | - |  |  |
| vvunid16ml5 | Hungary, *Vitis vinifera* | OQ931219 | OQ974170 | - | - | OQ973992 | - |  |  |
| vvunid16ml9 | Hungary, *Vitis vinifera* | OQ931131 | OQ974082 | - | OQ973739 | - | - |  |  |
| vvunid16rp3 | Hungary, *Vitis vinifera* | OQ931079 | OQ974031 | OQ973629 | OQ973740 | OQ973993 | - |  |  |
| vvunid16rp8 | Hungary, *Vitis vinifera* | OQ931080 | OQ974032 | OQ973630 | OQ973741 | OQ973994 | OQ973828 |  |  |
| vvunid16yl1 | Hungary, *Vitis vinifera* | OQ931128 | OQ974079 | OQ973620 | OQ973730 | OQ973981 | - |  |  |
| vvunid16yl10 | Hungary, *Vitis vinifera* | OQ931129 | OQ974080 | OQ973622 | OQ973732 | OQ973983 | - |  |  |
| vvunid16yl13 | Hungary, *Vitis vinifera* | OQ931217 | OQ974168 | - | - | OQ973984 | - |  |  |
| vvunid16yl14 | Hungary, *Vitis vinifera* | OQ931075 | OQ974027 | OQ973623 | OQ973733 | OQ973985 | - |  |  |
| vvunid16yl16 | Hungary, *Vitis vinifera* | OQ931146 | OQ974097 | OQ973624 | OQ973734 | OQ973986 | OQ973825 |  |  |
| vvunid16yl17 | Hungary, *Vitis vinifera* | OQ931076 | OQ974028 | OQ973625 | OQ973735 | OQ973987 | OQ973826 |  |  |
| vvunid16yl19 | Hungary, *Vitis vinifera* | OQ931170 | OQ974121 | OQ973626 | OQ973736 | OQ973988 | - |  |  |
| vvunid16yl20 | Hungary, *Vitis vinifera* | OQ931077 | OQ974029 | - | OQ973737 | OQ973989 | OQ973827 |  |  |
| vvunid16yl8 | Hungary, *Vitis vinifera* | OQ931078 | OQ974030 | OQ973621 | OQ973731 | OQ973982 | OQ973824 |  |  |
| vvunid17ml3 | Hungary, *Vitis vinifera* | OQ931134 | OQ974085 | - | OQ973746 | - | - |  |  |
| vvunid17ml5 | Hungary, *Vitis vinifera* | OQ931135 | OQ974086 | OQ973635 | OQ973747 | OQ973999 | - |  |  |
| vvunid17yl1 | Hungary, *Vitis vinifera* | OQ931132 | OQ974083 | OQ973631 | OQ973742 | OQ973995 | - |  |  |
| vvunid17yl2 | Hungary, *Vitis vinifera* | OQ931220 | OQ974171 | - | - | - | - |  |  |
| Supplementary Table 1. Isolates used in this study with reference isolates *sensu* Woudenberg et al. [23]. | | | | | | | | | |
| **Species, strain number** | **Origin** | **GenBank accession numbers** | | | | | | | |
|  |  | ITS | RPB2 | ALTA1 | endoPG | OPA10-2 | KOG1058 | TEF1 | GAPDH |
| vvunid17yl3 | Hungary, *Vitis vinifera* | OQ931133 | OQ974084 | OQ973632 | OQ973743 | OQ973996 | - |  |  |
| vvunid17yl8 | Hungary, *Vitis vinifera* | OQ931081 | OQ974033 | OQ973633 | OQ973744 | OQ973997 | - |  |  |
| vvunid17yl9 | Hungary, *Vitis vinifera* | OQ931082 | OQ974034 | OQ973634 | OQ973745 | OQ973998 | OQ973829 |  |  |
| vvunid6b13 | Hungary, *Vitis vinifera* | OQ931196 | OQ974147 | - | OQ973753 | OQ973875 | - |  |  |
| vvunid6b17 | Hungary, *Vitis vinifera* | OQ931197 | OQ974148 | OQ973517 | OQ973754 | OQ973876 | - |  |  |
| vvunid6b6 | Hungary, *Vitis vinifera* | OQ931055 | OQ974006 | OQ973513 | OQ973640 | OQ973847 | OQ973790 |  |  |
| vvunid6b7 | Hungary, *Vitis vinifera* | OQ931198 | OQ974149 | OQ973514 | OQ973752 | OQ973874 | - |  |  |
| vvunid6b8 | Hungary, *Vitis vinifera* | OQ931093 | OQ974045 | OQ973515 | - | OQ973848 | - |  |  |
| vvunid6ml10 | Hungary, *Vitis vinifera* | OQ931148 | OQ974099 | OQ973524 | OQ973672 | OQ973879 | OQ973792 |  |  |
| vvunid6ml13 | Hungary, *Vitis vinifera* | OQ931149 | OQ974100 | OQ973525 | OQ973673 | OQ973880 | OQ973793 |  |  |
| vvunid6ml14 | Hungary, *Vitis vinifera* | OQ931150 | OQ974101 | OQ973526 | OQ973674 | OQ973881 | - |  |  |
| vvunid6ml15 | Hungary, *Vitis vinifera* | OQ931199 | OQ974150 | OQ973527 | OQ973756 | OQ973882 | - |  |  |
| vvunid6ml3 | Hungary, *Vitis vinifera* | OQ931095 | OQ974047 | OQ973520 | - | OQ973851 | - |  |  |
| vvunid6ml4 | Hungary, *Vitis vinifera* | OQ931200 | OQ974151 | OQ973521 | OQ973755 | OQ973877 | - |  |  |
| vvunid6ml6 | Hungary, *Vitis vinifera* | OQ931056 | OQ974007 | OQ973522 | OQ973641 | OQ973852 | OQ973791 |  |  |
| vvunid6ml9 | Hungary, *Vitis vinifera* | OQ931096 | OQ974048 | OQ973523 | - | OQ973878 | - |  |  |
| vvunid6rp12 | Hungary, *Vitis vinifera* | OQ931097 | OQ974049 | OQ973530 | - | OQ973885 | - |  |  |
| vvunid6rp27 | Hungary, *Vitis vinifera* | OQ931057 | OQ974008 | OQ973531 | OQ973642 | OQ973853 | - |  |  |
| vvunid6rp28 | Hungary, *Vitis vinifera* | OQ931058 | OQ974009 | OQ973532 | OQ973643 | OQ973854 | - |  |  |
| vvunid6rp3 | Hungary, *Vitis vinifera* | OQ931201 | OQ974152 | OQ973528 | OQ973757 | OQ973883 | - |  |  |
| vvunid6rp4 | Hungary, *Vitis vinifera* | OQ931098 | OQ974050 | OQ973529 | - | OQ973884 | OQ973794 |  |  |
| vvunid6yl1 | Hungary, *Vitis vinifera* | OQ931094 | OQ974046 | OQ973518 | - | OQ973849 | - |  |  |
| vvunid7ml4 | Hungary, *Vitis vinifera* | OQ931059 | OQ974010 | OQ973538 | OQ973644 | OQ973891 | - |  |  |
| vvunid7rp10 | Hungary, *Vitis vinifera* | OQ931101 | OQ974053 | OQ973539 | - | OQ973892 | OQ973797 |  |  |
| vvunid7yl10 | Hungary, *Vitis vinifera* | OQ931099 | OQ974051 | OQ973536 | - | OQ973889 | OQ973796 |  |  |
| vvunid7yl2 | Hungary, *Vitis vinifera* | OQ931100 | OQ974052 | OQ973533 | - | OQ973887 | OQ973795 |  |  |
| vvunid7yl6 | Hungary, *Vitis vinifera* | OQ931202 | OQ974153 | OQ973535 | OQ973759 | OQ973888 | - |  |  |
| vvunid8b1 | Hungary, *Vitis vinifera* | OQ931102 | OQ974054 | OQ973540 | - | OQ973893 | OQ973798 |  |  |
| Supplementary Table 1. Isolates used in this study with reference isolates *sensu* Woudenberg et al. [23]. | | | | | | | | | |
| **Species, strain number** | **Origin** | **GenBank accession numbers** | | | | | | | |
|  |  | ITS | RPB2 | ALTA1 | endoPG | OPA10-2 | KOG1058 | TEF1 | GAPDH |
| vvunid8b4 | Hungary, *Vitis vinifera* | OQ931151 | OQ974102 | OQ973541 | OQ973676 | OQ973894 | OQ973799 |  |  |
| vvunid8ml2 | Hungary, *Vitis vinifera* | OQ931060 | OQ974011 | OQ973545 | OQ973645 | OQ973899 | - |  |  |
| vvunid8rp4 | Hungary, *Vitis vinifera* | OQ931061 | OQ974012 | OQ973547 | OQ973646 | OQ973901 | OQ973803 |  |  |
| vvunid8yl6 | Hungary, *Vitis vinifera* | OQ931203 | OQ974154 | - | OQ973764 | OQ973898 | - |  |  |
| ***Alternaria arborescens* SC** |  |  |  |  |  |  |  |  |  |
| CBS 105.24 | Unknown, *Solanum tuberosum* | KP124393 | KP124863 | KP123941 | KP124097 | KP124706 |  | KP125171 | KP124245 |
| CBS 105.49 | Italy, contaminant blood culture | KP124396 | KP124866 | KP123944 | KP124100 | KP124709 |  | KP125174 | KP124248 |
| CBS 108.41 | Unknown, wood | KP124394 | KP124864 | KP123942 | KP124098 | KP124098 |  | KP125172 | KP124246 |
| CBS 109730 | USA, *Solanum lycopersicum* | KP124399 | KP124869 | KP123946 | KP124103 | KP124713 |  | KP125177 | KP124251 |
| CBS 112633 | South Africa, *Malus domestica* | KP124400 | KP124870 | KP123947 | KP124104 | KP124714 |  | KP125178 | KP124252 |
| CBS 113.41 | Unknown, *Schizanthus* sp. | KP124395 | KP124865 | KP123943 | KP124099 | KP124708 |  | KP125173 | KP124247 |
| CBS 115189 | South Africa, *Citrus clementina* | KP124402 | KP124872 | KP123949 | KP124106 | KP124716 |  | KP125180 | KP124254 |
| CBS 115516 | South Africa, *Malus domestica* | KP124403 | KP124873 | KP123950 | KP124107 | KP124717 |  | KP125181 | KP124255 |
| CBS 115517 | South Africa, *Malus domestica* | KP124404 | KP124874 | KP123951 | KP124108 | KP124718 |  | KP125182 | KP124256 |
| CBS 116329 | Germany, *Malus domestica* | KP124405 | KP124875 | KP123952 | KP124109 | KP124719 |  | KP125183 | KP124257 |
| CBS 117587 | Netherlands, *Brassica* sp. | KP124406 | KP124876 | KP123953 | KP124110 | KP124720 |  | KP125184 | KP124258 |
| CBS 118389 | Japan, *Pyrus pyrifolia* | KP124407 | KP124877 | KP123954 | KP124111 | KP124721 |  | KP125185 | KP124259 |
| CBS 123235 | Denmark, human toenail | KP124410 | KP124880 | KP123957 | KP124114 | KP124724 |  | KP125188 | KP124261 |
| CBS 123266 | Denmark, human toenail | KP124411 | KP124881 | KP123958 | KP124115 | KP124725 |  | KP125189 | KP124262 |
| CBS 123267 | Denmark, human nail | KP124412 | KP124882 | KP123959 | KP124116 | KP124726 |  | KP125190 | KP124263 |
| CBS 124274 | Denmark, *Prunus* sp. | KP124413 | - | KP123960 | KP124117 | KP124727 |  | KP125191 | KP124264 |
| CBS 124281 | Denmark, *Triticum* sp. | KP124414 | KP124883 | KP123961 | KP124118 | KP124728 |  | KP125192 | KP124265 |
| CBS 124282 | Denmark, *Hordeum vulgare* | KP124415 | KP124884 | KP123962 | KP124119 | KP124729 |  | KP125193 | KP124266 |
| CBS 124283 | Russia, *Oryza* sp. | KP124416 | KP124885 | KP123963 | KP124120 | KP124730 |  | KP125194 | KP124267 |
| CBS 126.60 | UK, wood | KP124397 | KP124867 | JQ646390 | KP124101 | KP124710 |  | KP125175 | KP124249 |
| CBS 127263 | Mexico, human nasal infection | KP124417 | KP124886 | KP123964 | KP124121 | KP124731 |  | KP125195 | KP124268 |
| CBS 750.68 | France, *Phaseolus vulgaris* | KP124398 | KP124868 | KP123945 | KP124102 | KP124711 |  | KP125176 | KP124250 |
| CPC 25266 | Austria, *Pyrus* sp. | KP124418 | KP124887 | KP123965 | KP124122 | KP124732 |  | KP125196 | KP124269 |
| Supplementary Table 1. Isolates used in this study with reference isolates *sensu* Woudenberg et al. [23]. | | | | | | | | | |
| **Species, strain number** | **Origin** | **GenBank accession numbers** | | | | | | | |
|  |  | ITS | RPB2 | ALTA1 | endoPG | OPA10-2 | KOG1058 | TEF1 | GAPDH |
| CBS 101.13 (*A. geophila*) | Switzerland, peat soil | KP124392 | KP124862 | KP123940 | KP124096 | KP124705 | - | KP125170 | KP124244 |
| CBS 119545 (*A. senecionicola*) | New Zealand, *Senecio skirrhodon* | KP124409 | KP124879 | KP123956 | KP124113 | KP124723 | - | KP125187 | KP124260 |
| CBS 119544 (*A. cerealis*) | New Zealand, *Avena sativa* | MH863062 | KP124878 | KP123955 | - | KP124722 | - | KP125186 | KP124112 |
| vvchar5rp4 | Hungary, *Vitis vinifera* | OQ931181 | OQ974132 | OQ973510 | OQ973670 | OQ973872 | OQ973789 |  |  |
| vvchar5rp9 | Hungary, *Vitis vinifera* | OQ931182 | OQ974133 | OQ973512 | OQ973751 | OQ973873 | - |  |  |
| vvchar5yl1 | Hungary, *Vitis vinifera* | OQ931178 | OQ974129 | OQ973502 | OQ973666 | OQ973869 | - |  |  |
| vvchar5yl10 | Hungary, *Vitis vinifera* | OQ931179 | OQ974130 | OQ973506 | OQ973669 | OQ973871 | - |  |  |
| vvchar5yl2 | Hungary, *Vitis vinifera* | OQ931083 | OQ974035 | OQ973503 | OQ973667 | OQ973843 | OQ973787 |  |  |
| vvchar5yl9 | Hungary, *Vitis vinifera* | OQ931180 | OQ974131 | OQ973505 | OQ973668 | OQ973870 | - |  |  |
| vvlean4ml1 | Hungary, *Vitis vinifera* | OQ931173 | OQ974124 | OQ973491 | OQ973657 | OQ973861 | - |  |  |
| vvlean4ml11 | Hungary, *Vitis vinifera* | OQ931174 | OQ974125 | OQ973495 | OQ973660 | OQ973864 | - |  |  |
| vvlean4ml12 | Hungary, *Vitis vinifera* | OQ931175 | OQ974126 | OQ973496 | OQ973661 | OQ973865 | - |  |  |
| vvlean4ml3 | Hungary, *Vitis vinifera* | OQ931088 | OQ974040 | - | - | OQ973836 | - |  |  |
| vvlean4ml8 | Hungary, *Vitis vinifera* | OQ931176 | OQ974127 | OQ973492 | OQ973658 | OQ973862 | - |  |  |
| vvlean4ml9 | Hungary, *Vitis vinifera* | OQ931177 | OQ974128 | OQ973493 | OQ973659 | OQ973863 | - |  |  |
| vvmerl3ml7 | Hungary, *Vitis vinifera* | OQ931172 | OQ974123 | OQ973487 | OQ973656 | OQ973859 | - |  |  |
| vvpinn2rp14 | Hungary, *Vitis vinifera* | OQ931171 | OQ974122 | OQ973483 | OQ973654 | OQ973857 | - |  |  |
| vvunid12b1 | Hungary, *Vitis vinifera* | OQ931191 | OQ974142 | OQ973570 | OQ973775 | OQ973926 | OQ973812 |  |  |
| vvunid14yl11 | Hungary, *Vitis vinifera* | OQ931192 | OQ974143 | OQ973596 | OQ973779 | OQ973955 | OQ973819 |  |  |
| vvunid6yl6 | Hungary, *Vitis vinifera* | OQ931084 | OQ974036 | OQ973519 | OQ973671 | OQ973850 | - |  |  |
| vvunid7yl1 | Hungary, *Vitis vinifera* | OQ931183 | OQ974134 | - | OQ973758 | OQ973886 | - |  |  |
| vvunid7yl11 | Hungary, *Vitis vinifera* | OQ931184 | OQ974135 | OQ973537 | OQ973760 | OQ973890 | - |  |  |
| vvunid7yl3 | Hungary, *Vitis vinifera* | OQ931085 | OQ974037 | OQ973534 | OQ973675 | OQ973855 | - |  |  |
| vvunid8ml7 | Hungary, *Vitis vinifera* | OQ931188 | OQ974139 | OQ973546 | OQ973765 | OQ973900 | - |  |  |
| vvunid8rp10 | Hungary, *Vitis vinifera* | OQ931189 | OQ974140 | OQ973549 | OQ973767 | OQ973903 | - |  |  |
| vvunid8rp5 | Hungary, *Vitis vinifera* | OQ931190 | OQ974141 | OQ973548 | OQ973766 | OQ973902 | OQ973804 |  |  |
| vvunid8yl1 | Hungary, *Vitis vinifera* | OQ931185 | OQ974136 | OQ973542 | OQ973761 | OQ973895 | OQ973800 |  |  |
| vvunid8yl2 | Hungary, *Vitis vinifera* | OQ931186 | OQ974137 | OQ973543 | OQ973762 | OQ973896 | OQ973801 |  |  |
| Supplementary Table 1. Isolates used in this study with reference isolates *sensu* Woudenberg et al. [23]. | | | | | | | | | |
| **Species, strain number** | **Origin** | **GenBank accession numbers** | | | | | | | |
|  |  | ITS | RPB2 | ALTA1 | endoPG | OPA10-2 | KOG1058 | TEF1 | GAPDH |
| vvunid8yl4 | Hungary, *Vitis vinifera* | OQ931187 | OQ974138 | OQ973544 | OQ973763 | OQ973897 | OQ973802 |  |  |
| ***Alternaria burnsii*** |  |  |  |  |  |  |  |  |  |
| CBS 107.38 | India, *Cuminum cyminum* | KP124420 | KP124889 | KP123967 | KP124124 | KP124734 |  | KP125198 | JQ646305 |
| CBS 108.27 | Unknown, *Gomphrena globosa* | KC584236 | KC584468 | KP123850 | KP123997 | KP124605 |  | KC584727 | KC584162 |
| CBS 110.50 | Mozambique, *Gossypium sp.* | KP124421 | KP124890 | KP123968 | KP124125 | KP124735 |  | KP125199 | KP124271 |
| CBS 118816 | India, *Rhizophora mucronata* | KP124423 | KP124892 | KP123970 | KP124127 | KP124737 |  | KP125201 | KP124273 |
| CBS 118817 | India, *Tinospora cordifolia* | KP124424 | KP124893 | KP123971 | KP124128 | KP124738 |  | KP125202 | KP124274 |
| CBS 130264 | India, human sputum | KP124425 | KP124894 | KP123972 | KP124129 | KP124739 |  | KP125203 | KP124275 |
| CBS 879.95 | UK, Sorghum sp. | KP124422 | KP124891 | KP123969 | KP124126 | KP124736 |  | KP125200 | KP124272 |
| ***Alternaria betae-kenyensis*** |  |  |  |  |  |  |  |  |  |
| CBS 118810 | Kenya, *Beta vulgaris* var. *cicla* | KP124419 | KP124888 | KP123966 | KP124123 | KP124733 |  | KP125197 | KP124270 |
| CBS 107.38 | India, *Cuminum cyminum* | KP124420 | KP124889 | KP123967 | KP124124 | KP124734 |  | KP125198 | JQ646305 |
| CBS 110.50 | Mozambique, *Gossypium* sp. | KP124421 | KP124890 | KP123968 | KP124125 | KP124735 |  | KP125199 | KP124271 |
| CBS 118816 | India, *Rhizophora mucronata* | KP124423 | KP124892 | KP123970 | KP124127 | KP124737 |  | KP125201 | KP124273 |
| CBS 118817 | India, *Tinospora cordifolia* | KP124424 | KP124893 | KP123971 | KP124128 | KP124738 |  | KP125202 | KP124274 |
| CBS 130264 | India, human sputum | KP124425 | KP124894 | KP123972 | KP124129 | KP124739 |  | KP125203 | KP124275 |
| CBS 879.95 | UK, *Sorghum* sp. | KP124422 | KP124891 | KP123969 | KP124126 | KP124736 |  | KP125200 | KP124272 |
| ***Alternaria_dauci*** |  |  |  |  |  |  |  |  |  |
| CBS 117097 | USA, *Daucus carota* | KC584192 | KC584392 | KJ718678 | - | - |  | - | KC584111 |
| ***Alternaria eichhorniae*** |  |  |  |  |  |  |  |  |  |
| CBS 119778 | Indonesia, *Eichhornia crassipes* | KP124426 | KP124896 | - | KP124131 | KP124741 |  | KP125205 | KP124277 |
| CBS 489.92 | India, *Eichhornia crassipes* | KC146356 | KP124895 | KP123973 | KP124130 | KP124740 |  | KP125204 | KP124276 |
| ***Alternaria gaisen*** |  |  |  |  |  |  |  |  |  |
| CBS 118488 | Japan, *Pyrus pyrifolia* | KP124427 | KP124897 | KP123975 | KP124132 | KP124743 |  | KP125206 | KP124278 |
| CBS 632.93 | Japan, *Pyrus pyrifolia* | KC584197 | KC584399 | KP123974 | AY295033 | KP124742 |  | KC584658 | KC584116 |
| CPC 25268 | Portugal, unknown | KP124428 | KP124898 | KP123976 | KP124133 | KP124133 |  | KP125207 | KP124279 |
| ***Alternaria gossypina*** |  |  |  |  |  |  |  |  |  |
| CBS 100.23 | Unknown, *Malus domestica* | KP124429 | KP124899 | KP123977 | KP124134 | KP124745 |  | KP125208 | KP124280 |
| Supplementary Table 1. Isolates used in this study with reference isolates *sensu* Woudenberg et al. [23]. | | | | | | | | | |
| **Species, strain number** | **Origin** | **GenBank accession numbers** | | | | | | | |
|  |  | ITS | RPB2 | ALTA1 | endoPG | OPA10-2 | KOG1058 | TEF1 | GAPDH |
| CBS 102597 | USA, *Minneola tangelo* | KP124432 | KP124902 | KP123978 | KP124137 | KP124748 |  | KP125211 | KP124281 |
| CBS 102601 | Colombia, *Minneola tangelo* | KP124433 | KP124903 | KP123979 | KP124138 | KP124749 |  | KP125212 | KP124282 |
| CBS 104.32 | Zimbabwe, *Gossypium* sp. | KP124430 | KP124900 | JQ646395 | KP124135 | KP124746 |  | KP125209 | JQ646312 |
| CBS 107.36 | Indonesia, soil | KP124431 | KP124901 | JQ646393 | KP124136 | KP124747 |  | KP125210 | JQ646310 |
| ***Alternaria iridiaustralis*** |  |  |  |  |  |  |  |  |  |
| CBS 118404 | New Zealand, *Iris* sp. | KP124434 | KP124904 | KP123980 | KP124139 | KP124750 |  | KP125213 | KP124283 |
| CBS 118486 | Australia, *Iris* sp. | KP124435 | KP124905 | KP123981 | KP124140 | KP124751 |  | KP125214 | KP124284 |
| CBS 118487 | Australia, *Iris* sp. | KP124436 | KP124906 | KP123982 | KP124141 | KP124752 |  | KP125215 | KP124285 |
| ***Alternaria jacinthicola*** |  |  |  |  |  |  |  |  |  |
| CBS 133751 | Mali, *Eichhornia crassipes* | KP124438 | KP124908 | KP123984 | KP124143 | KP124754 |  | KP125217 | KP124287 |
| CBS 878.95 | Mauritius, *Arachis hypogaea* | KP124437 | KP124907 | KP123983 | KP124142 | KP124753 |  | KP125216 | KP124286 |
| CPC 25267 | Unknown*, Cucumis melo* var. *inodorus* | KP124439 | KP124909 | KP123985 | KP124144 | KP124755 |  | KP125218 | KP124288 |
| ***Alternaria longipes*** |  |  |  |  |  |  |  |  |  |
| CBS 113.35 | Unknown, *Nicotiana tabacum* | KP124440 | KP124910 | KP123986 | KP124145 | KP124756 |  | KP125219 | KP124289 |
| CBS 121332 | USA, *Nicotiana tabacum* | KP124443 | KP125227 | KP123989 | KP124149 | KP124760 |  | KP125227 | KP124292 |
| CBS 121333 | USA, *Nicotiana tabacum* | KP124444 | KP124914 | KP123990 | KP124150 | KP124761 |  | KP125223 | KP124293 |
| CBS 539.94 | USA, *Nicotiana tabacum* | KP124441 | KP124911 | KP123987 | KP124146 | KP124757 |  | KP125220 | KP124290 |
| CBS 540.94 | USA*, Nicotiana tabacum* | AY278835 | KC584409 | AY563304 | KP124147 | KP124758 |  | KC584667 | AY278811 |
| CBS 917.96 | USA, *Nicotiana tabacum* | KP124442 | KP124912 | KP123988 | KP124148 | KP124759 |  | KP125226 | KP124291 |
| ***Alternaria macrospora*** |  |  |  |  |  |  |  |  |  |
| CBS 117228 | USA, *Gossypium barbadense* | KC584204 | KC584410 | KJ718702 | - | - |  | - | KC584124 |
| ***Alternaria perpunctulata*** |  |  |  |  |  |  |  |  |  |
| CBS 115267 | USA, *Alternanthera philoxeroides* | KC584210 | KC584418 | - | - | - |  | KC584676 | KC584129 |
| ***Alternaria porri*** |  |  |  |  |  |  |  |  |  |
| CBS 116698 | USA, *Allium cepa* | - | KC584421 | KJ718726 | - | - |  | KC584679 | KC584132 |
| ***Alternaria pseudorostrata*** |  |  |  |  |  |  |  |  |  |
| CBS 119411 | USA, *Euphorbia pulcherrima* | NR136014 | KC584422 | - | - | - |  | - | - |
|  |  |  |  |  |  |  |  |  |  |
| Supplementary Table 1. Isolates used in this study with reference isolates *sensu* Woudenberg et al. [23]. | | | | | | | | | |
| **Species, strain number** | **Origin** | **GenBank accession numbers** | | | | | | | |
|  |  | ITS | RPB2 | ALTA1 | endoPG | OPA10-2 | KOG1058 | TEF1 | GAPDH |
| ***Alternaria solani*** |  |  |  |  |  |  |  |  |  |
| CBS 116651 | USA, *Solanum tuberosum* | KC584217 | KC584430 | - | - | - |  | - | KC584139 |
| ***Alternaria tagetica*** |  |  |  |  |  |  |  |  |  |
| CBS 479.81 | United Kingdom, *Tagetes erecta* | KC584221 | KC584434 | - | - | - |  | - | KC584143 |
| ***Alternaria tomato*** |  |  |  |  |  |  |  |  |  |
| CBS 114.35 | Unknown, *Solanum lycopersicum* | KP124446 | KP124916 | KP123992 | KP124152 | KP124763 |  | KP125225 | KP124295 |
| CBS 103.30 | Unknown, *Solanum lycopersicum* | KP124445 | KP124915 | KP123991 | KP124151 | KP124762 |  | KP125224 | KP124294 |
|  |  |  |  |  |  |  |  |  |  |
|  |  |  |  |  |  |  |  |  |  |
|  |  |  |  |  |  |  |  |  |  |
|  |  |  |  |  |  |  |  |  |  |
|  |  |  |  |  |  |  |  |  |  |
|  |  |  |  |  |  |  |  |  |  |
|  |  |  |  |  |  |  |  |  |  |
|  |  |  |  |  |  |  |  |  |  |
