# Supplementary material for: Untargeted metabolomic analyses support the main phylogenetic groups of the common plant-associated Alternaria fungi isolated from grapevine (Vitis vinifera)
Source: Sci Rep. 2023 Nov 7;13:19298. doi: 10.1038/s41598-023-46020-3 (PMC10630412; doi:10.1038/s41598-023-46020-3)
Supplement: Supplementary file 12 — Supplementary Table 2. [file 41598_2023_46020_MOESM12_ESM.docx]

| **Isolate name** | **Plant part** | **Cultivar** | **Plant protection** | **Sampling date** | **Geographical location** |
| --- | --- | --- | --- | --- | --- |
| vvfurm1ml2 | mature leaf | Furmint | conventional | 16.08.2019 | 47.923015, 20.413456 |
| vvfurm1yl1 | young leaf |  |  |  |  |
| vvpinn2b7 | berry | Pinot Noir | conventional | 16.08.2019 | 47.850528, 20.346972 |
| vvpinn2rp14 | rachis/pedicel |  |  |  |  |
| vvmerl3b7 | berry | Merlot | conventional | 21.08.2019 | 47.867073, 20.383386 |
| vvmerl3ml5 | mature leaf |  |  |  |  |
| vvmerl3ml7 |  |  |  |  |  |
| vvmerl3ml10 |  |  |  |  |  |
| vvmerl3rp1 | rachis/pedicel |  |  |  |  |
| vvmerl3yl3 | young leaf |  |  |  |  |
| vvlean4ml1 | mature leaf | Leányka | conventional | 21.08.2019 | 47.868441, 20.384952 |
| vvlean4ml3 |  |  |  |  |  |
| vvlean4ml8 |  |  |  |  |  |
| vvlean4ml9 |  |  |  |  |  |
| vvlean4ml10 |  |  |  |  |  |
| vvlean4ml11 |  |  |  |  |  |
| vvlean4ml12 |  |  |  |  |  |
| vvlean4yl4 | young leaf |  |  |  |  |
| vvchar5b1 | berry | Chardonnay | conventional | 21.08.2019 | 47.864751, 20.381422 |
| vvchar5b2 |  |  |  |  |  |
| vvchar5b11 |  |  |  |  |  |
| vvchar5b12 |  |  |  |  |  |
| vvchar5b13 |  |  |  |  |  |
| vvchar5rp1 | rachis/pedicel |  |  |  |  |
| vvchar5rp2 |  |  |  |  |  |
| vvchar5rp4 |  |  |  |  |  |
| vvchar5rp8 |  |  |  |  |  |
| vvchar5rp9 |  |  |  |  |  |
| vvchar5yl1 | young leaf |  |  |  |  |
| vvchar5yl2 |  |  |  |  |  |
| vvchar5yl3 |  |  |  |  |  |
| vvchar5yl9 |  |  |  |  |  |
| vvchar5yl10 |  |  |  |  |  |
| vvchar5yl16 |  |  |  |  |  |
| vvchar5yl17 |  |  |  |  |  |
| vvunid6b6 | berry | Unknown | untreated/abandoned | 22.08.2019 | 47.917889, 20.358778 |
| **Isolate name** | **Plant part** | **Cultivar** | **Plant protection** | **Sampling date** | **Geographical location** |
| vvunid6b7 | berry | Unknown | untreated/abandoned | 22.08.2019 | 47.917889, 20.358778 |
| vvunid6b8 |  |  |  |  |  |
| vvunid6b13 |  |  |  |  |  |
| vvunid6b17 |  |  |  |  |  |
| vvunid6ml3 | mature leaf |  |  |  |  |
| vvunid6ml4 |  |  |  |  |  |
| vvunid6ml6 |  |  |  |  |  |
| vvunid6ml9 |  |  |  |  |  |
| vvunid6ml10 |  |  |  |  |  |
| vvunid6ml13 |  |  |  |  |  |
| vvunid6ml14 |  |  |  |  |  |
| vvunid6ml15 |  |  |  |  |  |
| vvunid6rp3 | rachis/pedicel |  |  |  |  |
| vvunid6rp4 |  |  |  |  |  |
| vvunid6rp12 |  |  |  |  |  |
| vvunid6rp27 |  |  |  |  |  |
| vvunid6rp28 |  |  |  |  |  |
| vvunid6yl1 | young leaf |  |  |  |  |
| vvunid6yl6 |  |  |  |  |  |
| vvunid7ml4 | mature leaf | Unknown | conventional | 22.08.2019 | 47.919750, 20.356556 |
| vvunid7rp10 | rachis/pedicel |  |  |  |  |
| vvunid7yl1 | young leaf |  |  |  |  |
| vvunid7yl2 |  |  |  |  |  |
| vvunid7yl3 |  |  |  |  |  |
| vvunid7yl6 |  |  |  |  |  |
| vvunid7yl10 |  |  |  |  |  |
| vvunid7yl11 |  |  |  |  |  |
| vvunid8b1 | berry | Unknown | conventional | 29.08.2019 | 47.927750, 20.344389 |
| vvunid8b4 |  |  |  |  |  |
| vvunid8ml2 | mature leaf |  |  |  |  |
| vvunid8ml7 |  |  |  |  |  |
| vvunid8rp4 | rachis/pedicel |  |  |  |  |
| vvunid8rp5 |  |  |  |  |  |
| vvunid8rp10 |  |  |  |  |  |
| vvunid8yl1 | young leaf |  |  |  |  |
| vvunid8yl2 |  |  |  |  |  |
| **Isolate name** | **Plant part** | **Cultivar** | **Plant protection** | **Sampling date** | **Geographical location** |
| vvunid8yl4 | young leaf | Unknown | conventional | 29.08.2019 | 47.927750, 20.344389 |
| vvunid8yl6 |  |  |  |  |  |
| vvkada9ml2 | mature leaf | Kadarka | organic | 29.08.2019 | 47.924500, 20.352806 |
| vvkada9ml4 |  |  |  |  |  |
| vvkada9ml5 |  |  |  |  |  |
| vvkada9ml7 |  |  |  |  |  |
| vvkada9yl4 | young leaf |  |  |  |  |
| vvkada9yl8 |  |  |  |  |  |
| vvkada9yl11 |  |  |  |  |  |
| vvkada9yl12 |  |  |  |  |  |
| vvunid10ml1 | mature leaf | Unknown | organic | 28.08.2019 | 47.905168, 20.352119 |
| vvunid10ml2 |  |  |  |  |  |
| vvunid10ml7 |  |  |  |  |  |
| vvunid10ml8 |  |  |  |  |  |
| vvunid10rp1 | rachis/pedicel |  |  |  |  |
| vvunid10rp3 |  |  |  |  |  |
| vvunid10rp5 |  |  |  |  |  |
| vvunid11b1 | berry | Unknown | untreated | 05.09.2019 | 47.838611, 20.401250 |
| vvunid11ml1 | mature leaf |  |  |  |  |
| vvunid11rp6 | rachis/pedicel |  |  |  |  |
| vvunid11rp10 |  |  |  |  |  |
| vvunid11rp11 |  |  |  |  |  |
| vvunid11rp16 |  |  |  |  |  |
| vvunid11rp22 |  |  |  |  |  |
| vvunid12b1 | berry | Unknown | untreated/abandoned | 05.09.2019 | 47.836500, 20.400611 |
| vvunid12ml1 | mature leaf |  |  |  |  |
| vvunid12ml2 |  |  |  |  |  |
| vvunid12ml8 |  |  |  |  |  |
| vvunid12ml9 |  |  |  |  |  |
| vvunid12ml12 |  |  |  |  |  |
| vvunid12ml15 |  |  |  |  |  |
| vvunid12ml16 |  |  |  |  |  |
| vvunid12ml17 |  |  |  |  |  |
| vvunid12rp1 | rachis/pedicel |  |  |  |  |
| vvunid12rp2 |  |  |  |  |  |
| vvunid12yl1 | young leaf |  |  |  |  |
| **Isolate name** | **Plant part** | **Cultivar** | **Plant protection** | **Sampling date** | **Geographical location** |
| vvunid12yl2 | young leaf | Unknown | untreated/abandoned | 05.09.2019 | 47.836500, 20.400611 |
| vvunid12yl3 |  |  |  |  |  |
| vvunid12yl10 |  |  |  |  |  |
| vvunid13ml1 | mature leaf | Unknown | untreated/abandoned | 05.09.2019 | 47.838583, 20.401639 |
| vvunid13ml2 |  |  |  |  |  |
| vvunid13rp4 | rachis/pedicel |  |  |  |  |
| vvunid13rp5 |  |  |  |  |  |
| vvunid13rp10 |  |  |  |  |  |
| vvunid13rp13 |  |  |  |  |  |
| vvunid13yl1 | young leaf |  |  |  |  |
| vvunid13yl6 |  |  |  |  |  |
| vvunid14ml7 | mature leaf | Unknown | untreated/abandoned | 05.09.2019 | 7.836500, 20.400333 |
| vvunid14ml10 |  |  |  |  |  |
| vvunid14ml11 |  |  |  |  |  |
| vvunid14ml12 |  |  |  |  |  |
| vvunid14ml13 |  |  |  |  |  |
| vvunid14yl1 | young leaf |  |  |  |  |
| vvunid14yl4 |  |  |  |  |  |
| vvunid14yl5 |  |  |  |  |  |
| vvunid14yl7 |  |  |  |  |  |
| vvunid14yl11 |  |  |  |  |  |
| vvunid14yl10 |  |  |  |  |  |
| vvunid14yl12 |  |  |  |  |  |
| vvunid14yl14 |  |  |  |  |  |
| vvunid15b2 | berry | Unknown | untreated/abandoned | 12.09.2019 | 47.840750, 20.422611 |
| vvunid15b3 |  |  |  |  |  |
| vvunid15b10 |  |  |  |  |  |
| vvunid15ml1 | mature leaf |  |  |  |  |
| vvunid15ml7 |  |  |  |  |  |
| vvunid15rp1 | rachis/pedicel |  |  |  |  |
| vvunid15rp2 |  |  |  |  |  |
| vvunid15rp3 |  |  |  |  |  |
| vvunid15rp14 |  |  |  |  |  |
| vvunid15yl1 | young leaf |  |  |  |  |
| vvunid15yl2 |  |  |  |  |  |
| vvunid15yl3 |  |  |  |  |  |
| **Isolate name** | **Plant part** | **Cultivar** | **Plant protection** | **Sampling date** | **Geographical location** |
| vvunid15yl7 | young leaf | Unknown | untreated/abandoned | 12.09.2019 | 47.840750, 20.422611 |
| vvunid15yl10 |  |  |  |  |  |
| vvunid15yl12 |  |  |  |  |  |
| vvunid15yl15 |  |  |  |  |  |
| vvunid16b1 | berry | Unknown | untreated/abandoned | 12.09.2019 | 47.841306, 20.424056 |
| vvunid16b10 |  |  |  |  |  |
| vvunid16ml1 | mature leaf |  |  |  |  |
| vvunid16ml2 |  |  |  |  |  |
| vvunid16ml5 |  |  |  |  |  |
| vvunid16ml9 |  |  |  |  |  |
| vvunid16rp3 | rachis/pedicel |  |  |  |  |
| vvunid16rp8 |  |  |  |  |  |
| vvunid16yl1 | young leaf |  |  |  |  |
| vvunid16yl8 |  |  |  |  |  |
| vvunid16yl10 |  |  |  |  |  |
| vvunid16yl13 |  |  |  |  |  |
| vvunid16yl14 |  |  |  |  |  |
| vvunid16yl16 |  |  |  |  |  |
| vvunid16yl17 |  |  |  |  |  |
| vvunid16yl19 |  |  |  |  |  |
| vvunid16yl20 |  |  |  |  |  |
| vvunid17ml3 | mature leaf | Unknown | untreated/abandoned | 30.09.2019 | 47.920444, 20.355306 |
| vvunid17ml5 |  |  |  |  |  |
| vvunid17yl1 | young leaf |  |  |  |  |
| vvunid17yl2 |  |  |  |  |  |
| vvunid17yl3 |  |  |  |  |  |
| vvunid17yl8 |  |  |  |  |  |
| vvunid17yl9 |  |  |  |  |  |
