# Supplementary material for: Untargeted metabolomic analyses support the main phylogenetic groups of the common plant-associated Alternaria fungi isolated from grapevine (Vitis vinifera)
Source: Sci Rep. 2023 Nov 7;13:19298. doi: 10.1038/s41598-023-46020-3 (PMC10630412; doi:10.1038/s41598-023-46020-3)
Supplement: Supplementary file 13 — Supplementary Table 3. [file 41598_2023_46020_MOESM13_ESM.docx]

| **Primer name** | **Primer sequence 5’ – 3’** | **Annealing temperature (°C)** | **Reference** |
| --- | --- | --- | --- |
| ITS1f  ITS4 | CTGGTCATTTAGAGGAAGTAA  TCCTCCGCTTATTGATATGC | 52 | 40, 41 |
| RPB2-6F  fRPB2-7cR | TGGGGKWTGGTYTGYCCTGC  CCCATRGCTTGYTTRCCCAT | 52 | 42 |
| Alt-for  Alt-rev | ATGCAGTTCACCACCATCGC  ACGAGGGTGAYGTAGGCGTC | 65 | 16 |
| PG3  PG2b | TACCATGGTTCTTTCCGA  GAGAATTCRCARTCRTCYTGRTT | 61 | 17 |
| OPA10-2R  OPA10-2L | GATTCGCAGCAGGGAAACTA  TCGCAGTAAGACACATTCTACG | 53 | 17 |
| KOG1058F2  KOG1058R2 | GAGTCACGTTAYCGCASC  TGGCTKACGGARACG | 53 | 23 |
| Alt4-for  Alt4-rev | ATGCAGTTCACCACCATCGCYTC  ACGAGGGTGAYGTAGGCGTCRG | 61 | 43 |
